# A Multi-Center, Randomized, Controlled, Pivotal Study to Assess the Safety and Efficacy of a Selective Cytopheretic Device in Patients with Acute Kidney Injury

**DOI:** 10.1371/journal.pone.0132482

**Published:** 2015-08-05

**Authors:** James A. Tumlin, Claude M. Galphin, Ashita J. Tolwani, Micah R. Chan, Anitha Vijayan, Kevin Finkel, Balazs Szamosfalvi, Devasmita Dev, J. Ricardo DaSilva, Brad C. Astor, Alexander S. Yevzlin, H. David Humes, SCD Investigator Group

**Affiliations:** 1 Department of Medicine, UT College of Medicine, University of Tennessee, 960 East Third Street, Suite 100, Chattanooga, TN, 37403, United States of America; 2 Department of Medicine, UAB School of Medicine, University of Alabama, 1720 2nd Ave. S. FOT 1203, Birmingham, AL, 35294–3412, United States of America; 3 Department of Medicine, University of Wisconsin, UW Med Fndtn. Centennial Bldg. 5148 MFCB, 1685 Highland Ave., Madison, WI, 53705–2281, United States of America; 4 Washington University School of Medicine, 660 S Euclid Ave., St Louis, MO, 63110, United States of America; 5 UT Health Science Center, University of Texas, 6410 Fannin St., Ste. 606, Houston, TX, 77030, United States of America; 6 Henry Ford Health System, Henry Ford Hospital, CFP-509, 2799 West Grand Blvd., Detroit, MI, 48202–2608, United States of America; 7 Dallas VA Medical Center, 4500 S. Lancaster Rd., Dallas, TX, 75216, United States of America; 8 CytoPherx, Inc., 401 W. Morgan Rd., Ann Arbor, MI, 48108, United States of America; 9 Department of Population Health Sciences, University of Wisconsin, Health Sciences Learning Center, 750 Highland Ave., Madison, WI, 53705, United States of America; 10 Department of Medicine, University of Michigan, 4520 MSRB I, Box 0651, 1150 W. Medical Center Drive, Ann Arbor, MI, 48109, United States of America; Ichan School of Medicine at Mount Sinai, UNITED STATES

## Abstract

**Objective:**

Acute kidney injury (AKI) is a highly morbid condition in critically ill patients that is associated with high mortality. Previous clinical studies have demonstrated the safety and efficacy of the Selective Cytopheretic Device (SCD) in the treatment of AKI requiring continuous renal replacement therapy in the intensive care unit (ICU).

**Design, Setting, Patients:**

A randomized, controlled trial of 134 ICU patients with AKI, 69 received continuous renal replacement therapy (CRRT) alone and 65 received SCD therapy.

**Results:**

No significant difference in 60-day mortality was observed between the treated (27/69; 39%) and control patients (21/59; 36%, with six patients lost to follow up) in the intention to treat (ITT) analysis. Of the 19 SCD subjects (CRRT+SCD) and 31 control subjects (CRRT alone) who maintained a post-filter ionized calcium (iCa) level in the protocol’s recommended range (≤ 0.4mmol/L) for greater or equal to 90% of the therapy time, 60-day mortality was 16% (3/19) in the SCD group compared to 41% (11/27) in the CRRT alone group (p = 0.11). Dialysis dependency showed a borderline statistically significant difference between the SCD treated versus control CRRT alone patients maintained for ≥ 90% of the treatment in the protocol’s recommended (r) iCa target range of ≤ 0.4 mmol/L with values of, 0% (0/16) and 25% (4/16), respectively (P = 0.10). When the riCa treated and control subgroups were compared for a composite index of 60 day mortality and dialysis dependency, the percentage of SCD treated subjects was 16% versus 58% in the control subjects (p<0.01). The incidence of serious adverse events did not differ between the treated (45/69; 65%) and control groups (40/65; 63%; p = 0·86).

**Conclusion:**

SCD therapy may improve mortality and reduce dialysis dependency in a tightly controlled regional hypocalcaemic environment in the perfusion circuit.

**Trial Registration:**

ClinicalTrials.gov NCT01400893 http://clinicaltrials.gov/ct2/show/NCT01400893

## Introduction

Acute kidney injury (AKI) is a highly morbid condition in critically ill patients with mortality rates exceeding 50% [1–5]. Although dialysis removes waste products and corrects fluid imbalance, it does not perform the absorptive, metabolic, endocrine, and immunologic functions of normal renal tubule cells. As a result, AKI promotes a systemic inflammatory response syndrome (SIRS) which results in systemic microvascular damage and, if severe, multi-organ dysfunction [6, 7]. Activated circulating leukocytes play a central role in this process [8]. Leukocytes, especially neutrophils, are major contributors to the pathogenesis and progression of many inflammatory disorders, including SIRS, sepsis, ischemia reperfusion injury, and acute respiratory distress syndrome (ARDS). Many therapeutic approaches are under investigation to limit the activation and tissue accumulation of leukocytes at sites of inflammation to minimize tissue destruction and disease progression [9–11].

The Selective Cytopheretic Device (SCD) is comprised of tubing, connectors, and a synthetic membrane cartridge that binds and deactivates leukocytes which are activated from an inflammatory process, and, when used in a continuous renal replacement extracorporeal circuit in the presence of regional citrate anticoagulation (RCA), modulates inflammation [12]. Previous clinical studies have demonstrated the safety and efficacy of the SCD in the treatment of AKI requiring continuous renal replacement therapy (CRRT) in the intensive care unit (ICU). In a Phase-IIa, prospective, single-arm, single-center study designed to evaluate the safety and device integrity of treatment with the SCD [13], 12 patients enrolled in the trial were compared with historical case-matched controls with respect to age and Sequential Organ Failure Assessment (SOFA) score. The mortality for the case-matched controls was 78%, whereas the mortality in the SCD treatment group was 22% (p = 0·03). Multivariate regression analysis identified treatment with SCD as the only significant variable affecting mortality. Mean total urine output in the 10 subjects receiving SCD treatment increased from a baseline of approximately 500 ml/d to more than 2,000 ml/d by day seven of treatment. No serious device related adverse events were reported. In a Phase-IIb, prospective, single-arm, multicenter US pilot study designed to evaluate the safety and efficacy of the SCD treatment on AKI requiring CRRT in the ICU [14]. The study enrolled 35 subjects. The average SOFA score of the patients was

11·3 ± 3·6. Death from any cause at Day 60 was 31·4%. Renal recovery, defined as dialysis independence, was observed in all of the surviving subjects at Day 60. The results of this pilot study indicated the potential for a substantial improvement in patient outcomes over standard of care therapy (CRRT alone), and prompted the design of the Phase IIIa trial described herein.

The primary objective of this study was to determine whether CRRT + SCD treatment, compared to CRRT alone, results in significantly improved all-cause mortality through Day 60. Secondary objectives included an assessment of renal replacement therapy (RRT) dependency at Day 60, mortality at Day 28, the number of ventilator free days (VFD) at Day 28, and the mortality of the subset of patients with severe sepsis at Day 60.

## Materials and Methods

This was a two-arm, randomized, open-label, controlled multi-center study and was performed under an FDA-approved investigational device exemption (IDE# G090189). This study and the clinical protocol are accessible at clinicaltrial.gov, ID# NCT 01400893. Each enrolling site had IRB approval to undertake this clinical investigation. The institutional review boards named below specifically approved this study, and all participants involved in the study signed informed consent.

### Center Name / IRB Type

University of Texas/Local, University of Wisconsin/Central WIRB (Western Institutional Review Board), University of Maryland/Local, Massachusetts General/Local, U of California Los Angeles/Local, Mount Sinai/Local, SERRI (Memorial)/Central WIRB, SERRI (Erlanger)/Local, Cleveland Clinic/Local, Northwestern University/Local, U of California San Diego (UCSD)/Local, U of Florida (Jacksonville)/Central WIRB, Virginia Commonwealth/Central WIRB, Medical U of S Carolina/Central WIRB, Beth Israel Deaconess/Local, U of Florida (Gainesville)/Central WIRB, University of Arizona/Central WIRB, Dallas VA/Local, Henry Ford Health System/Local, VA Medical Center Buffalo/Local, Washington University/Local, University of Mississippi/Local, U of Alabama Birmingham/Central IRB, INOVA Fairfax/Central WIRB, University of Iowa/Central WIRB, Albany Medical College/Central WIRB, (WNERTA) Western N England Renal and Transplant Associates /Local.

134 subjects were enrolled in 21 US medical centers. Patients receiving care in the ICU of each participating hospital were randomized to intensive care treatment for patients undergoing CRRT or CRRT + SCD. Any mode of CRRT was allowed (CVVH, CVVHD, and CVVHDF) following each participating clinical site’s protocol, with minimal effluent rate (dialysate and ultrafiltrate) of at least 25ml/Kg/h. The modality of CRRT was not expected to affect performance of the SCD. All CRRT was delivered via one pre-specified dialysis pump system (B. Braun Diapact CRRT System), and was supplied by CytoPherx, Inc. (Ann Arbor, MI)

Each participating clinical site used their established regional citrate anticoagulation (RCA) protocol for the CRRT and SCD circuits (Study Arm) and for the CRRT only (Control Arm). The recommended ionized calcium level (measured post SCD-ARF) in the CRRT and SCD-ARF blood circuit was specified to be between 0·25 and 0·4 mmol/L. Inclusion and exclusion criteria are described in Tables 1 and 2.

**Table 1:** Inclusion criteria.

1.	A patient, or legal representative, has signed a written informed consent form.
2.	Must be receiving medical care in an intensive care unit (e.g., ICU, MICU, SICU, CTICU, Trauma).
3.	Age 18 to 80 years.
4.	Females of child bearing potential who are not pregnant (confirmed by a negative serum pregnancy test) and not lactating if recently post-partum.
5.	Must be receiving and tolerating CRRT therapy for a minimum of 4 hours, but not longer than 24.
6.	Expected to remain in the ICU for at least 96 hours after evaluation for enrollment.
7.	A clinical diagnosis of ATN due to hemodynamic or toxic etiologies. ATN is defined as Acute Kidney Injury occurring in a setting of acute ischemic or nephrotoxic injury with oliguria (average <20 mL/hr) for >6–12 hours ***or*:** an increase in serum creatinine ≥2 mg/dL (≥1.5 mg/dL in females) over a period of ≤4 days. (Note: Prerenal, hepatorenal, vascular, interstitial, glomerular, and obstructive etiologies are excluded on clinical or other diagnostic grounds.)
8.	At least one non-renal organ failure (SOFA organ system score ≥2) or presence (proven or suspected) of sepsis.
9.	All patients must be able to tolerate regional citrate anticoagulation.

**Table 2:** Exclusion Criteria.

1.	Irreversible brain damage based on available historical and clinical information.
2.	Presence of a renal transplant at any time.
3.	Non-renal organ transplantation within six month of screening.
4.	Presence of preexisting advanced chronic renal failure (i.e., ESRD) requiring chronic renal replacement therapy prior to this episode of acute kidney injury.
5.	AKI occurring in the setting of burns, obstructive uropathy, allergic interstitial nephritis, acute or rapidly progressive glomerulonephritis, vasculitis, hemolytic-uremic syndrome, thrombotic thrombocytopenic purpura (TTP), malignant hypertension, scleroderma renal crisis, atheroembolism, functional or surgical nephrectomy, hepatorenal syndrome, cyclosporine or tacrolimus nephrotoxicity.
6.	Metastatic malignancy which is actively being treated or may be treated by chemotherapy or radiation during the subsequent three month period after study therapy.
7.	Chronic immunosuppression (e.g., HIV/AIDS, chronic glucocorticoid therapy >20 mg/day prednisone equivalent on a chronic basis). The acute use of glucocorticoids is permissible.
8.	Severe liver failure as documented by a Child-Pugh Liver Failure Score >12 (see Appendix F).
9.	Do Not Resuscitate Status (DNR).
10.	Comfort measures only
11.	Patient is moribund or for whom full supportive care is not indicated.
12.	Patient is not expected to survive 28 days because of an irreversible medical condition. (This is not restrictive to AKI, and may include situations such as the presence of irreversible brain damage, untreatable malignancy, inoperable life threatening condition, or any condition to which therapy is regarded as futile by the PI.)
13.	Any medical condition that the Investigator thinks may interfere with the study objectives.
14.	Physician refusal.
15.	Patient is a prisoner.
16.	Dry weight of >150 kg.
17.	More than one hemodialysis treatment during this hospital admission or prior to transfer from an outside hospital.
18.	Platelet count <30,000/mm^3^
19.	Concurrent enrollment in another interventional clinical trial. Patients enrolled in clinical trials where only measurements and/or samples are taken (NO TEST DEVICE OR TEST DRUG USED) are allowed to participate.
20.	Use of any other Investigational drug or device within the previous 30 days.

A centralized randomization system was established with 24-hour availability to provide a mechanism to randomize subjects and enroll them in the study. Once the patient met all eligibility criteria, including being on CRRT for a minimum of four hours, but no longer than 24 hours, and had signed informed consent, the subject was randomized in a 1:1 allocation utilizing a random permuted block design into either the control or treatment arm, stratified by study center and the presence of severe sepsis.

**Fig 1:**
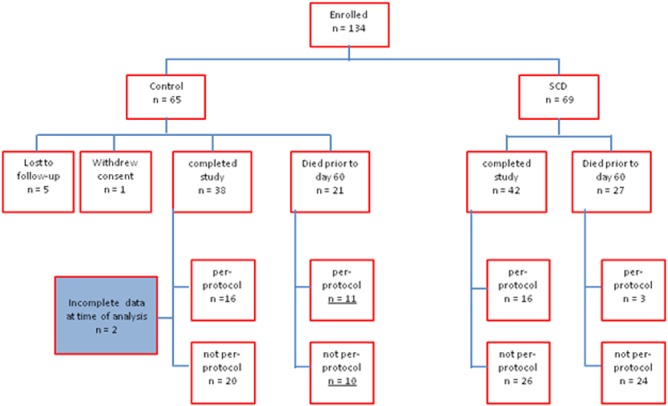
Flow chart of the disposition in all of the patients (N = 134) enrolled. For the purpose of statistical analysis ITT is defined as all control and all treatment, whereas mITT is defined as all control and all treatment with iCa at recommended range >90% of the time.

Patients randomized to the control arm received renal replacement therapy utilizing a CRRT pump system that is identical to that used for the SCD treatment, tubing and hemofilter provided by the sponsor (CytoPherx, Inc., Ann Arbor, MI). Anticoagulation of the system was accomplished using the institution’s protocol for citrate anticoagulation with a target range for intra-circuit ionized calcium of 0·25–0·40 mmol/L. All patients in the control arm were required to use citrate as an anticoagulant. Patients randomized to the study treatment arm received renal replacement therapy identical to the control arm, plus the SCD (Fig 1). The SCD was placed in the CRRT blood circuit as detailed (S1 Fig).

An overall two-sided 0·05 level of significance at 80% power was used to calculate a sample size of 344 patients, assuming a mortality rate of 50% for control and 35% for the treatment groups [1–5]. An interim analysis for sample size re-estimation was planned at the mid-point of enrollment (172 subjects).

The primary outcome was all-cause mortality through Day 60. Secondary outcomes included an assessment of renal replacement therapy (RRT) dependency at Day 60, mortality at Day 28, the number of ventilator free days (VFD) at Day 28, and the mortality of the subset of patients with severe sepsis at Day 60. Several exploratory biomarkers were also compared between the control and treatment groups, including urine output, serum levels of elastase, IL-6, and IL-10, and total absolute white blood cell, neutrophil, and platelet counts throughout treatment.

Fisher exact tests were used to compare the primary and outcomes, and chi-square tests were used for the occurrence of adverse events across randomized treatment groups for the intention to treat (ITT) population as well as the modified intention to treat (mITT) population. ITT was defined as all control and all treatment, whereas mITT is defined as all control and all treatment with iCa at recommended range >90% of the time. Kaplan-Meier curves and log-rank tests were used to compare the time to mortality across treatment groups. Differences between treatment groups in continuous outcomes (e.g., urine output, white blood cell count, number of neutrophils, elastase) over time were assessed with repeated measures linear regression models, including an interaction term to estimate the difference in change over time (i.e. slope) between groups. These models excluded the baseline (i.e., pre-treatment) values. Statistical analyses were performed with Stata/MP 12·1 (www.stata.com). Two-sided p-values of <0·05 were considered statistically significant.

## Results

The trial was initiated on September 8, 2011. During the second quarter of the enrollment period, a national calcium shortage occurred in the US. Due to the reliance of the SCD on a narrow intra-circuit ionized calcium range for it to function effectively and the concern that patients randomized to the SCD were not getting effective therapy, the interim analysis was performed early—after enrollment of 134 patients. Enrollment was paused on May 24, 2013, to assess the clinical impact of the calcium shortage on study endpoints. The shortage of calcium infusion solutions resulted in a tendency to minimize citrate infusion rates. Accordingly, the ionized calcium levels within the blood circuit tended to be above the recommended range of 0·25 to 0·40 mmol/L in the majority of the subjects. The study was terminated by the sponsor at the interim analysis because the SCD treatment was often outside the recommended iCa range, and therefore, resulted in ineffective therapy.

No significant differences were noted between the ITT or mITT control and treatment groups in terms of baseline characteristics Table 3. Of the 134 patients in the analysis, 69 received CRRT alone and 65 received SCD therapy (S1 Fig). The days on CRRT were similar in both groups with control and treatment groups averaging 5.4 ± 2.4 and 5.2 ± 2.1 days, respectively. No statistically significant difference was found between the treated and control patients of the ITT population, with a 60 day mortality of 39% (27/69) and 36% (21/59) respectively, with six patients in the control group lost to follow up, Table 4, (S2 Fig) (log-rank p-value = 0.23).

**Table 3:** Demographics of All Study Subjects.

	CRRT + SCD	CRRT Alone	Overall
	N = 69	N = 65	N = 134
Age (years)	57.2 ± 13.1 (69)	53.5 ± 14.7 (65)	55.4 ± 14.0 (134)
Sex			
Male	60.9% (42/69)	61.5% (40/65)	61.2% (82/134)
Female	39.1% (27/69)	38.5% (25/65)	38.8% (52/134)
Ethnicity			
Hispanic or Latino	4.3% (3/69)	3.1% (2/65)	3.7% (5/134)
Non-Hispanic or Latino	89.9% (62/69)	86.2% (56/65)	88.1% (118/134)
Unknown	5.8% (4/69)	10.8% (7/65)	8.2% (11/134)
Race			
American Indian or Alaska Native	0.0% (0/69)	0.0% (0/65)	0.0% (0/134)
Asian	0.0% (0/69)	0.0% (0/65)	0.0% (0/134)
Black/African American	21.7% (15/69)	21.5% (14/65)	21.6% (29/134)
Native Hawaiian or Other Pacific Islander	0.0% (0/69)	0.0% (0/65)	0.0% (0/134)
White/Caucasian	76.8% (53/69)	73.8% (48/65)	75.4% (101/134)
Unknown	0.0% (0/69)	1.5% (1/65)	0.7% (1/134)
Other	1.4% (1/69)	3.1% (2/65)	2.2% (3/134)
Body Weight (kg)	102.0 ± 23.1 (62)	98.8 ± 23.6 (56)	100.5 ± 23.3 (118)
SOFA Score	13.8 ± 3.2 (67)	13.2 ± 3.7 (64)	13.5 ± 3.4 (131)
Child Pugh Score	8.1 ± 1.6 (66)	8.1 ± 1.4 (62)	8.1 ± 1.5 (128)
Severe Sepsis Status			
Yes	65.2% (45/69)	69.2% (45/65)	67.2% (90/134)
No	34.8% (24/69)	30.8% (20/65)	32.8% (44/134)
Ventilator Status			
Yes	88.4% (61/69)	90.8% (59/65)	89.6% (120/134)
No	11.6% (8/69)	9.2% (6/65)	10.4% (14/134)
CRRT Modality			
CVVH	15.9% (11/69)	22.2% (14/63)	18.9% (25/132)
CVVHD	31.9% (22/69)	27.0% (17/63)	29.5% (39/132)
CVVHDF	52.2% (36/69)	49.2% (31/63)	50.8% (67/132)
Other	0.0% (0/69)	1.6% (1/63)	0.8% (1/132)
BUN (mg/dl)	43.2 ± 25.9 (69)	40.6 ± 29.7 (65)	42.9 ± 27.8 (134)
Creatinine (mg/dl)	2.98 ± 1.65 (69)	2.93 ± 1.70 (65)	2.96 ± 1.6 (134)

**Table 4:** 60 Day Mortality by Intention to Treat Analysis.

60 Day Mortality	CRRT + SCD	CRRT Alone	Overall
	N = 69	N = 65	N = 134
All Subjects Enrolled	100.0% (69/69)	100.0% (65/65)	100.0%(134/134)
Alive	61% (42/69)	64% (38/59*)	63% (80/128)
Dead	39% (27/69)	36% (21/59*)	38% (48/128)

* Does not include six subjects LTFU (002–003, 011–002, 004–007, 011–004, 013–002, 007–025).

The hazard ratio for mortality within the first 60 days associated with treatment was 1.32 (95% confidence interval [CI]: 0.74, 2.35).

There were no statistically significant differences in the secondary endpoints (RRT dependency at Day 60, mortality at Day 28, VFD at Day 28, and the mortality of the subset of patients with severe sepsis at Day 60) between the treatment and control group of the ITT population. Table 5 delineates the summary of site-reported serious adverse events (SAEs) using site-reported category and term for the ITT population. No statistically significant difference was found between the SAEs of the control and treatment groups. Furthermore, none of the SAEs were considered ‘definitely’ device related per the principal investigator. Overall adverse events did not differ between the treatment and control groups in the ITT analysis.

**Table 5:** Summary of Site-reported Serious Adverse Events (SAEs).

All Subjects N = 132*							
	CRRT + SCD		CRRT Alone			Total	
	N = 69		N = 63*			N = 132*	
Category	Ets	Pts	Ets	Pts	Fisher's Exact	Ets	Pts
		% (n/N)		% (n/N)	P-Value		% (n/N)
Total	80	65.2% (45/69)	71	63.5% (40/63)	0.857	151	64.4% (85/132)
Blood and lymphatic system disorders	9	11.6% (8/69)	4	4.8% (3/63)	0.212	13	8.3% (11/132)
Cardiac disorders	15	17.4% (12/69)	11	15.9% (10/63)	1.000	26	16.7% (22/132)
Gastrointestinal disorders	5	5.8% (4/69)	7	9.5% (6/63)	0.518	12	7.6% (10/132)
General disorders and administration site conditions	4	5.8% (4/69)	7	11.1% (7/63)	0.350	11	8.3% (11/132)
Infections and infestations	14	17.4% (12/69)	11	15.9% (10/63)	1.000	25	16.7% (22/132)
Injury, poisoning and procedural complications	1	1.4% (1/69)	0	0.0% (0/63)	1.000	1	0.8% (1/132)
Investigations	0	0.0% (0/69)	1	1.6% (1/63)	0.477	1	0.8% (1/132)
Metabolism and nutrition disorders	2	2.9% (2/69)	2	3.2% (2/63)	1.000	4	3.0% (4/132)
Musculoskeletal and connective tissue disorders	1	1.4% (1/69)	1	1.6% (1/63)	1.000	2	1.5% (2/132)
Nervous system disorders	6	7.2% (5/69)	1	1.6% (1/63)	0.211	7	4.5% (6/132)
Other	2	2.9% (2/69)	6	7.9% (5/63)	0.258	8	5.3% (7/132)
Psychiatric disorders	0	0.0% (0/69)	1	1.6% (1/63)	0.477	1	0.8% (1/132)
Renal and urinary disorders	1	1.4% (1/69)	3	4.8% (3/63)	0.348	4	3.0% (4/132)
Respiratory, thoracic and mediastinal disorders	13	14.5% (10/69)	10	15.9% (10/63)	1.000	23	15.2% (20/132)
Skin and subcutaneous tissue disorders	0	0.0% (0/69)	2	3.2% (2/63)	0.226	2	1.5% (2/132)
Vascular disorders	7	10.1% (7/69)	4	6.3% (4/63)	0.536	11	8.3% (11/132)

* Two subjects enrolled to CRRT alone arm (012–002, 003–015) however not treated.

The amount of time the subjects in both the control and treatment group were maintained in the recommended ionized calcium range (0·25–0·40 mmol/L), as specified in the study protocol, was substantially lower than expected. Of the 134 subjects enrolled in the SCD-003 protocol at the time of the interim analysis, 19 SCD subjects (CRRT+SCD) and 31 control subjects (CRRT alone) were maintained in the protocol’s recommended range (≤ 0·4mmol/L) for greater or equal to 90% of the therapy time. Table 6 and (S3 Fig) (log-rank p-value = 0.27) describe the mortality at Day 60 (primary endpoint) of the treated subjects which received the recommended ionized calcium (riCa). Mortality in this subset was 16% (3/19) in the SCD group compared to 41% (11/27) in the CRRT alone group, (p_exact_ = 0·11). The hazard ratio for mortality within 60 days for this subset was 0.46 (95% CI: 0.13, 1.65). This subset of subjects did not differ significantly in terms of age, gender, race, presence of sepsis, or mean SOFA score (Table 7) and did not differ in baseline characteristics from the non-riCa

**Table 6:** 60 Day Mortality of Subjects–Recommended Ionized Calcium Range (riCa).

60 Day Mortality	CRRT + SCD	CRRT Alone	Overall
riCa	N = 19	N = 27*	N = 46
Alive	84% (16/19)	59% (16/27)	70% (32/46)
Dead	16% (3/19)	41% (11/27)	30% (14/46)

* Three subjects LTFU.

**Table 7:** Baseline characteristics of the riCa and non-riCa vs. control subset of patients.

Potential confounding variable	CRRT Alone	CRRT + SCD	riCa SCD	Non-riCa SCD
	N = 57	N = 69	N = 19	N = 50
Age (mean)	54.4 ±14.7	58.2 ± 13.0	57.2 ±13.7	58.5 ± 12.8
% Female	38.4	39.1	31.6	42.0
% White	73.8	76.8	89.5	67.9
% Severe Sepsis	69.2	65.2	68.4	64.0
SOFA (mean)	11.8 ±4.1	12.7 ± 4.1	12.0 ±4.1	12.9 ± 4.1

The secondary endpoints of renal replacement therapy dependency at Day 60, mortality at Day 28, number of ventilator free days at Day 28 and mortality of the sub population of severe septic patients at Day 60 were analyzed. No statistical significance was shown except for the secondary endpoint of dialysis dependency. Dialysis dependency (Table 8) showed a borderline statistically significant difference between the patients maintained for ≥ 90% of the treatment in the protocol’s riCa target range of ≤ 0.4 mmol/L over those at ≤ 90% of the treatment duration, 0% (0/16) and 25% (4/16), respectively (P_exact_ = 0·10). The association of SCD treatment with mortality at Day 60 differed significantly by iCa protocol adherence (p-interaction = 0.03), as did the association of treatment with the composite outcome of 60-day mortality or dialysis dependency (p-interaction = 0.004). When the riCa treated and all (intention to treat) control subgroups were compared for this composite outcome, the percentage of SCD treated subjects was 16% versus 58% in the control subjects (p_exact_ = 0.01).

**Table 8:** Mortality or Dialysis Dependency at Day 60 –Recommended Ionized Calcium Range.

Mortality or Dialysis Dependency at Day 60 –Recommended Ionized Calcium Range	CRRT + SCD	CRRT Alone
N	3 / 19	15 / 26
p_exact_	0.01	

Several exploratory biomarkers were evaluated. Neither urine output, elastase, IL-6, and IL-10 levels, total white blood cell, neutrophil, and platelet counts throughout treatment differed in the entire 134 subject cohort. When the recommended ionized calcium (riCa) subpopulation was considered, a statistically significant difference was detected in several parameters: log urine output substantially increased (S4 Fig), white blood cell count (WBC) and neutrophil count diminished (S5 and S6 Figs), and elastase increased (S7 Fig) in the treatment vs. control group over time.

## Discussion

Inflammation plays a key role in acute and chronic organ dysfunction [9–11]. Acute organ dysfunction, including AKI, acute lung injury, acute myocardial infarction, and stroke is well correlated to the degree of inflammation. In this study we evaluated the safety and efficacy of a novel approach to the treatment of kidney failure developing from excessive dysregulated inflammation. A selective cytopheretic device, which immunomodulates activated circulating leukocytes in the presence of a citrate induced hypocalcemic environment within an extracorporeal blood circuit, including neutrophils and monocytes was compared to standard of care therapy with CRRT alone.

To understand the mechanism of action of this device on a profoundly difficult clinical disorder, a series of investigations were performed. Since AKI results in an acute inflammatory response state resulting in microvascular dysfunction in multiple organs [15], immunoflourescence microscopy of the SCD demonstrated adherent leukocytes on the outer surface of the membranes of the cartridge along the blood flow path within the extracorporeal circuit [13]. The sequestered leukocytes were dominated with neutrophils. (S8 Fig) The ability of leukocytes to bind to the outer walls of the hollow fiber membranes rather than the inner walls, which is the conventional blood flow path, was recognized to be due to the shear forces of blood flow. The sheer stress (SS) of blood along the outer wall of the membrane was near capillary SS of <1 dyne/cm2 compared to the SS of nearly 100 dyne/cm2 of blood when flowing along the inner conventional surfaces of the hollow fiber membranes. The role of citrate infusion in this device blood circuit is related to the effect of citrate to lower the ionized calcium (iCa) levels of blood to below 0·4mM, a level which inhibits the coagulation system of blood. This lower blood iCa also has an inhibitory effect on neutrophil activation [13], resulting in a simultaneously combination effect to sequester activated circulating leukocytes and alter the activity of the bound leukocytes. Further studies now suggest that the bound leukocytes were subsequently released back to the systemic circulation in a less inflammatory state [16]. Consequently, the membrane cartridge is referred to as a selective cytopheretic device (SCD) and in the presence of citrate anticoagulation, an immunomodulatory membrane device.

This clinical observation was previously evaluated using the SCD with citrate anticoagulation in a well-established porcine model of E. coli induced septic shock [12]. These studies demonstrated an ability of the SCD with citrate to lower systemic neutrophil activation, diminish aggregation of activated leukocytes in the lungs, decrease systemic capillary leak, preserve cardiac output (CO), ameliorate renal dysfunction and prolong survival time compared to various control groups [17]. Further experiments have suggested that the “catch and release” of activated neutrophils within the SCD promoted the activated neutrophil, which is in a delayed apoptotic state and has a longer life span, to revert back to a normal time to apoptosis and a normal life span despite the presence of a SIRS state. This observation is consistent with previous work that suggests that blocking calcium entry into a neutrophil activates the apoptotic pathway to programmed cell death [18].

The amount of time the subjects in both the control and treatment group were maintained in the recommended ionized calcium range (0·25–0·40 mmol/L), as specified in the study protocol, was substantially lower than expected. One reason for the deficiency in clinical trial execution to ensure the protocol’s riCa target range of ≤ 0·40 mmol/L is maintained could be attributed to the national shortage of injectable calcium. If the patient did not experience circuit clotting, the primary investigators’ emphasis of achieving and continuously maintaining the patient in the recommended ionized calcium range was not consistently adhered to. In addition, the injectable calcium shortage resulted in 9 of the 21 open clinical sites unable to enroll subjects due to low hospital inventories of injectable calcium. As a result, the study was terminated. The results of this study will be used to design a new clinical trial protocol intended to achieve a statistically significant difference in SCD therapy vs. CRRT alone on a death or dialysis dependence composite endpoint at 60 days in the treatment of AKI in the setting of critical illness.

The early termination of the study is a major limitation, but one that leads to a critical conclusion: that the SCD and a calcium concentration of <0·40 mmol/L are both necessary conditions for immunomodulation. The observation that, in those patients who had the prescribed iCa level >90% of the time on SCD, mortality improved from 41% to 16%, is clinically compelling. In addition, the observation, both in the pilot Phase II SCD trial [14], and the Phase IIIa study reported here, that no patient receiving appropriate SCD therapy was dialysis dependent at Day 60 is also compelling. Previous large prospective clinical studies in AKI with multiorgan dysfunction had a greater than 20% incidence of dialysis dependency of patients followed for 60 or more days [18, 19]. A sensitivity analysis for riCa in the targeted range was performed for 80%, 85%, and 90% of the treatment time. The mortality effect on SCD-treated patients was not observed when riCa treatment time was below 90% of that which was prescribed (data not shown).

Leukocytes play a key role in reperfusion injury after AKI [20–22], and the effect of the SCD to modulate excessive leukocyte activation most likely plays a critical role in the recovery of renal function after a substantive AKI event. The relationship of ongoing inflammation in the kidney after AKI and chronic progressive kidney disease and dialysis dependency has been demonstrated previously, and may explain the observation in this study that dialysis dependency was decreased for the SCD treated group [23, 24]. Furthermore, the significant divergence of serum elastase levels, as a specific neutrophil activity biomarker between the treated and control groups is also noteworthy. SCD therapy appears to maintain neutrophil activity late in the SIRS process compared to the compensatory anti-inflammatory response which follows the excessive systemic proinflammatory state in AKI and MOD [25]. Thus, the significant decrease in WBC and neutrophil counts, the difference in elastase, the rates of dialysis dependence, as well as the improvement in urine output over time corroborates the mechanistic role of leukocytes and inflammation on AKI previously published [12–15, 26]. The precise mechanism of action is being investigated with data suggesting a role of normalizing the delay in neutrophil apoptosis promoted by systemic inflammation.

Based on the results described above, one may ask: Is the trend toward improved survival in the target calcium group the result of low calcium alone or the SCD device, or both. In a post hoc analysis there was no difference between controls with calcium levels < .04 and controls with calcium levels > .04., suggesting that calcium level alone in the hemofilter is not a sufficient condition for a survival advantage (S9 Fig) (log-rank p-value = 0.85). In a post hoc analysis we also compared subjects treated with the SCD who achieved calcium levels < .04 with those treated with the SCD who achieved calcium levels > .04 (S9 Fig) (log-rank p-value = 0.03). This revealed a significant mortality benefit, suggesting that low ionized calcium in the presence of the SCD is a necessary and sufficient condition for better outcomes.

## Conclusion

ITT analysis did not demonstrate efficacy of the SCD with 60 day all cause mortality. Intra SCD calcium levels exceeding 0.4 mm/L were demonstrated in the majority of sites. Circuit ionized Ca++ levels < 0.4 mm/L alone did not alter 60 day all-cause mortality rates. Circuit Ca++ levels < 0.4 mm/L in combination with the SCD reduced 60 day all-cause mortality from 40.8% to 15.8% in a post-hoc analysis. While there is no evidence that SCD used in combination with CRRT improves patient survival currently, further studies examining the SCD device in more regulated perfusion circuit calcium environments are warranted.

## Supporting Information

S1 CONSORT Checklist CONSORT Checklist. (PDF)

S1 Fig Schematic of SCD in the CRRT circuit. (TIF)

S2 Fig Kaplan-Meier analysis of mortality at Day 60 for 134 patients by ITT analysis (log-rank p-value = 0·23). (TIF)

S3 Fig Kaplan-Meier analysis of mortality at Day 60 for those patients who received the recommended iCa dose >90% of the time when treated with the SCD (log-rank p-value = 0·27). (TIF)

S4 Fig Urine output of treated vs. control groups for those patients who received the recommended iCa dose of >90% of the time when treated with the SCD. (TIF)

S5 Fig WBC of treated vs. control groups for those patients who received the recommended iCa dose of >90% of the time when treated with the SCD. (TIF)

S6 Fig Percent Neutrophil count of treated vs. control groups for those patients who received the recommended iCa dose of >90% of the time when treated with the SCD. (TIF)

S7 Fig Elastase level (ng/ml) of treated vs. control groups for those patients who received the recommended iCa dose of >90% of the time when treated with the SCD. (TIF)

S8 Fig Kaplan-Meier curve of mortality at Day 60 of riCa and non-riCa subgroups in the Control patients (log-rank p-value = 0.85). (TIF)

S9 Fig Kaplan-Meier analysis of mortality at Day 60 for riCa and non-riCa subgroups in the SCD treated patients (log-rank p-value = 0.03). (TIF)

S1 Protocol SCD-003_protocol_v1.4. (PDF)
