# Supplementary figures and images for: A Multi-Center, Randomized, Controlled, Pivotal Study to Assess the Safety and Efficacy of a Selective Cytopheretic Device in Patients with Acute Kidney Injury
Source: PLoS One. 2015 Aug 5;10(8):e0132482. doi: 10.1371/journal.pone.0132482 (PMC4526678; doi:10.1371/journal.pone.0132482)

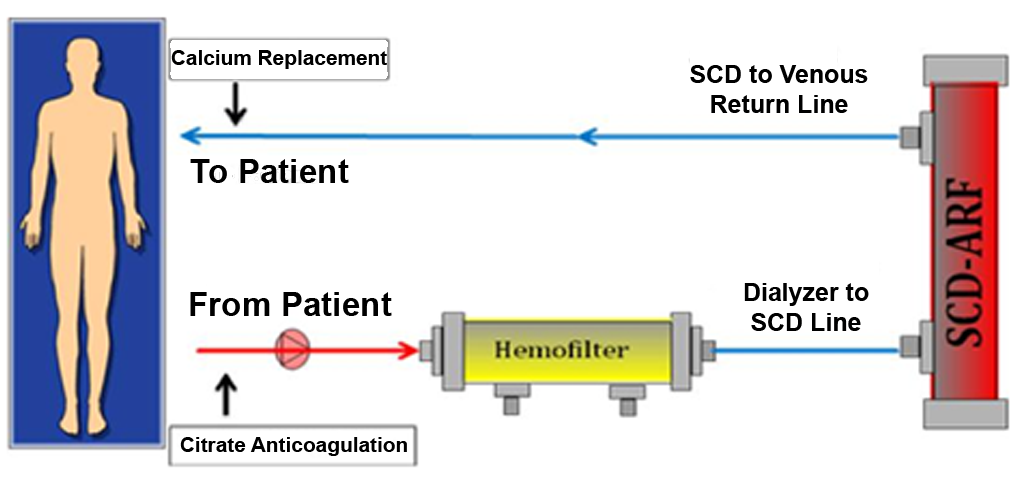

Supplement: S1 Fig — (TIF) [file pone.0132482.s002.tif]

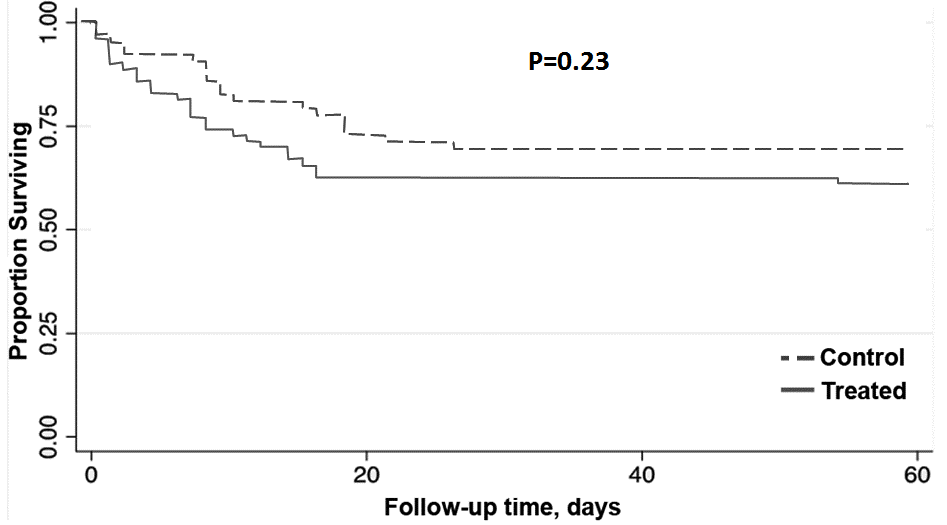

Supplement: S2 Fig — (TIF) [file pone.0132482.s003.tif]

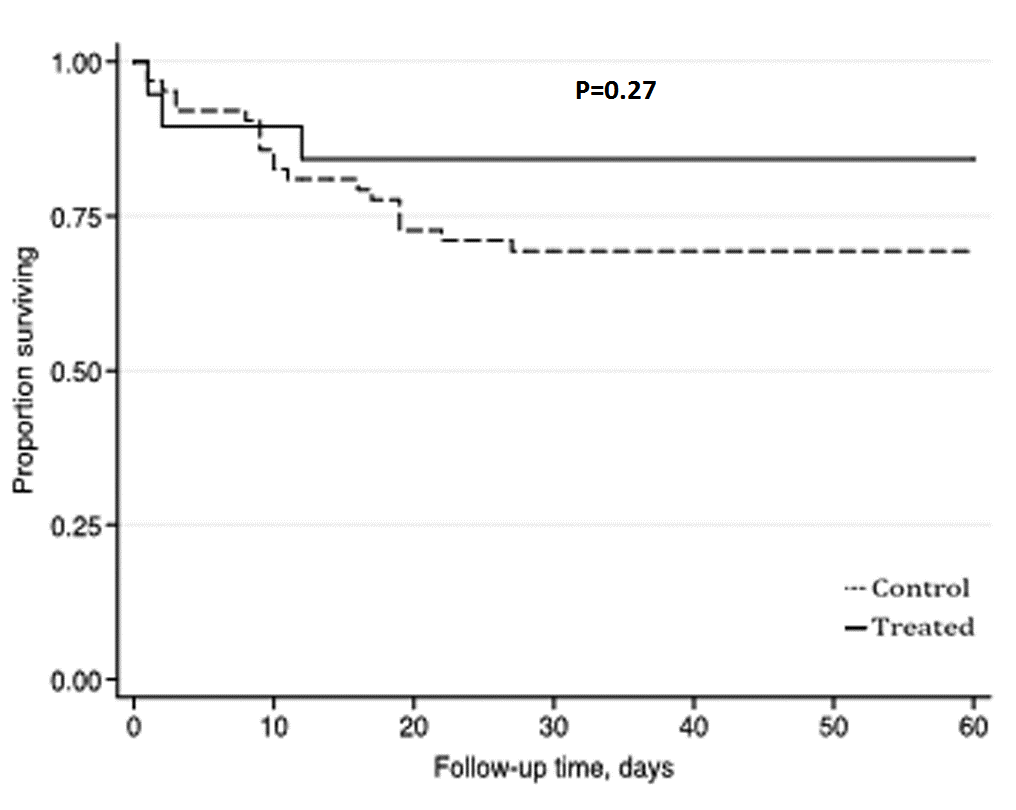

Supplement: S3 Fig — (TIF) [file pone.0132482.s004.tif]

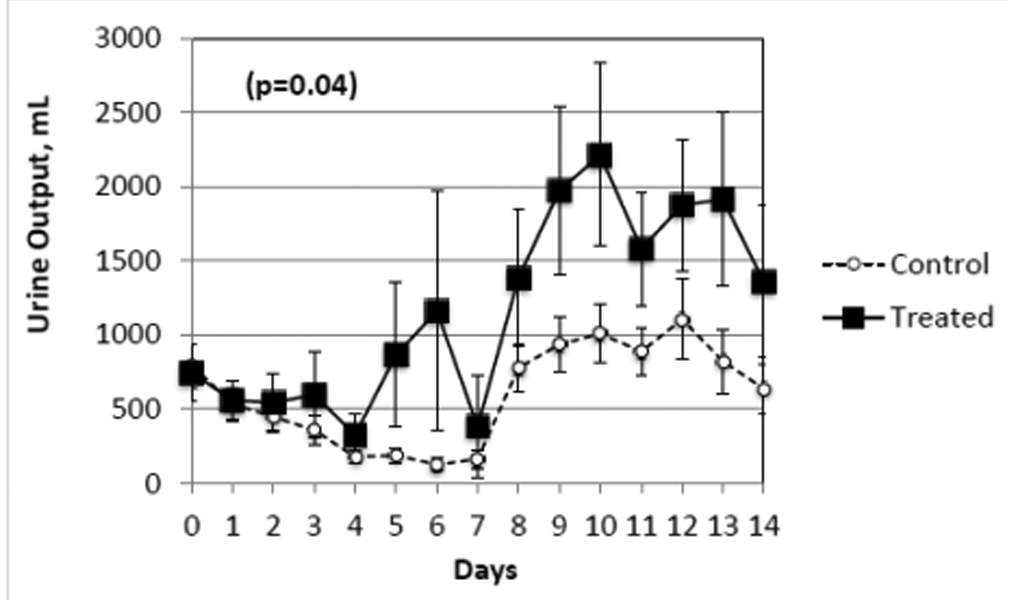

Supplement: S4 Fig — (TIF) [file pone.0132482.s005.tif]

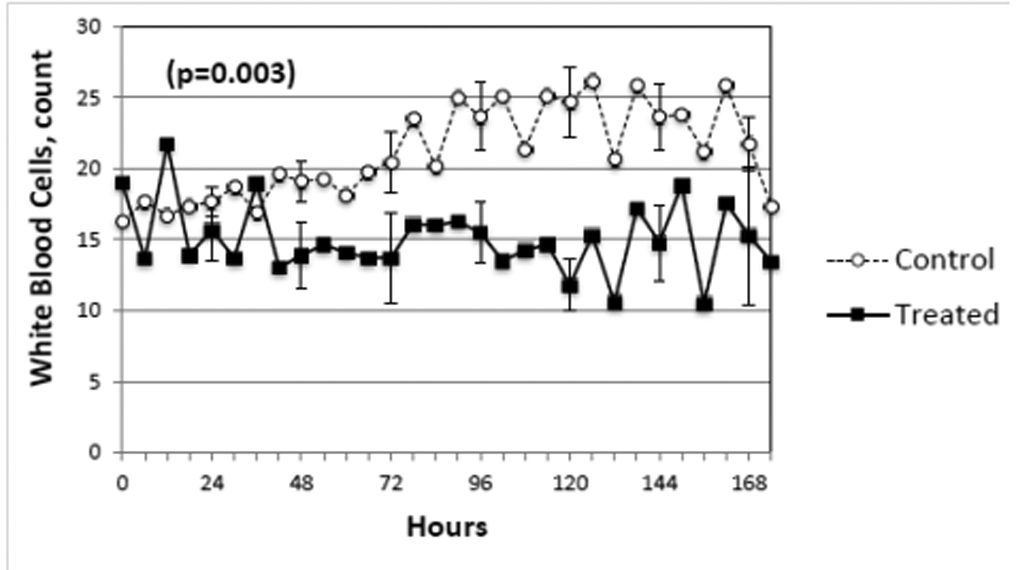

Supplement: S5 Fig — (TIF) [file pone.0132482.s006.tif]

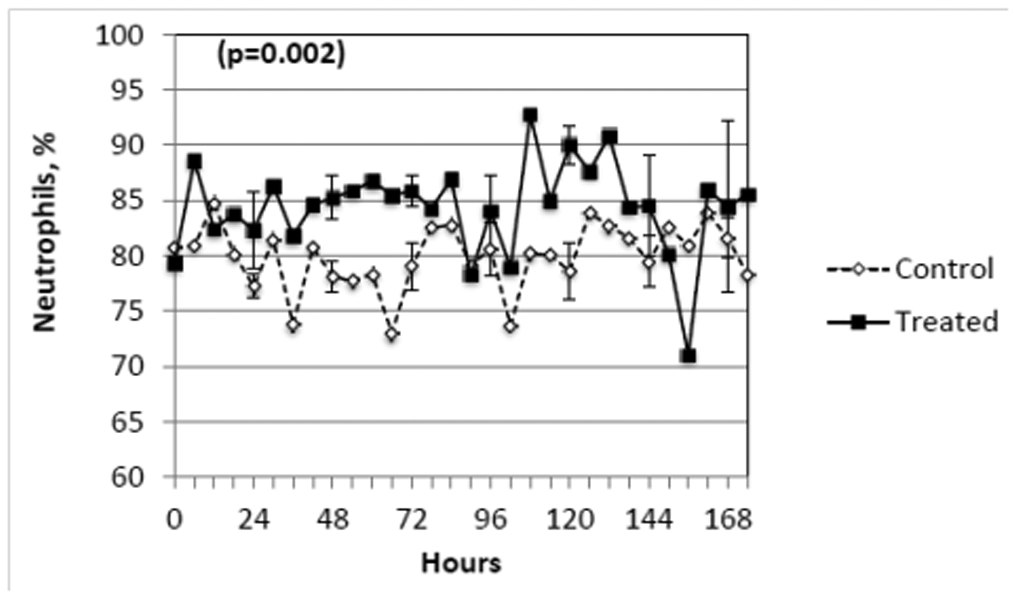

Supplement: S6 Fig — (TIF) [file pone.0132482.s007.tif]

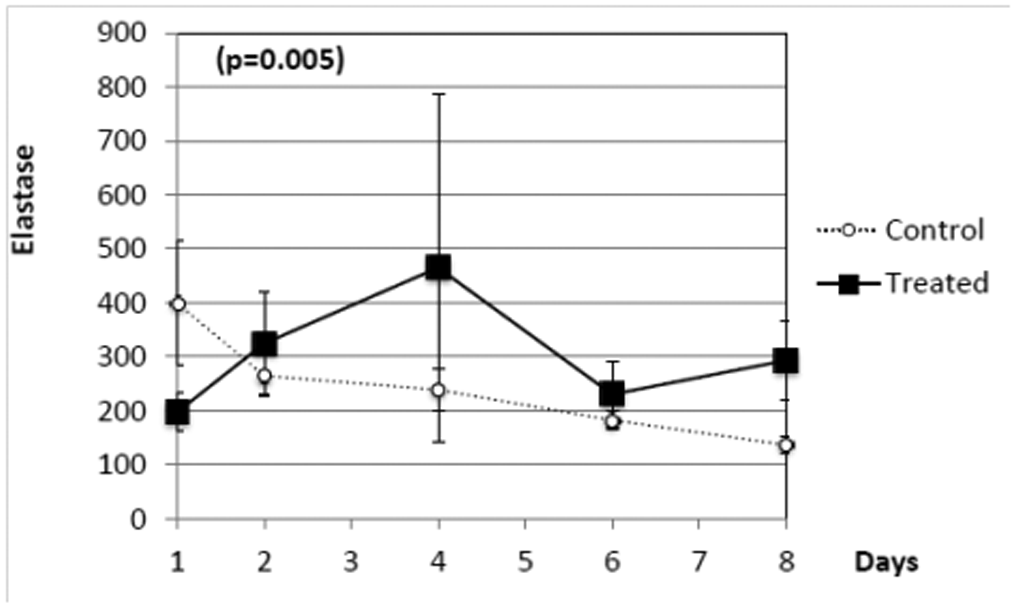

Supplement: S7 Fig — (TIF) [file pone.0132482.s008.tif]

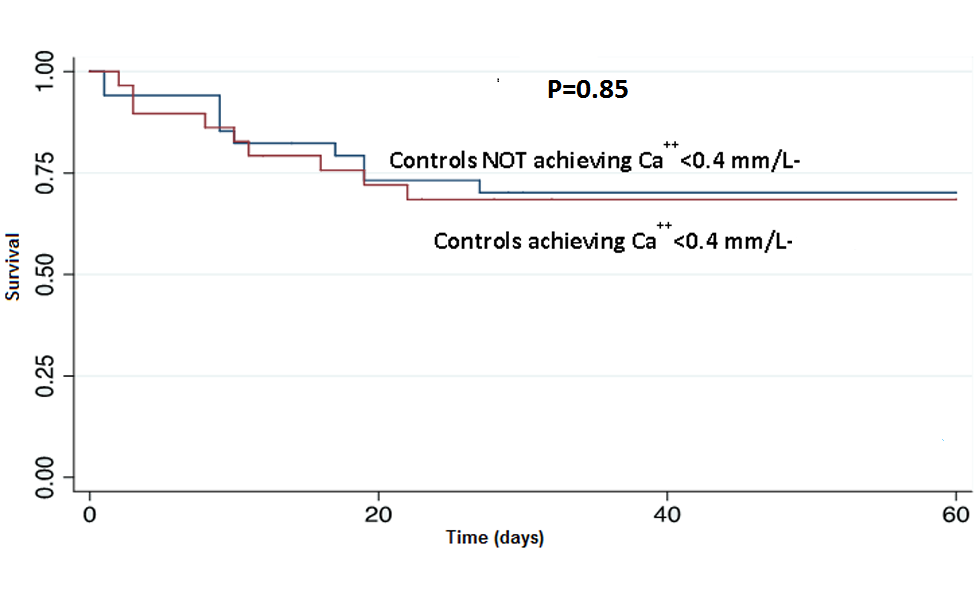

Supplement: S8 Fig — (TIF) [file pone.0132482.s009.tif]

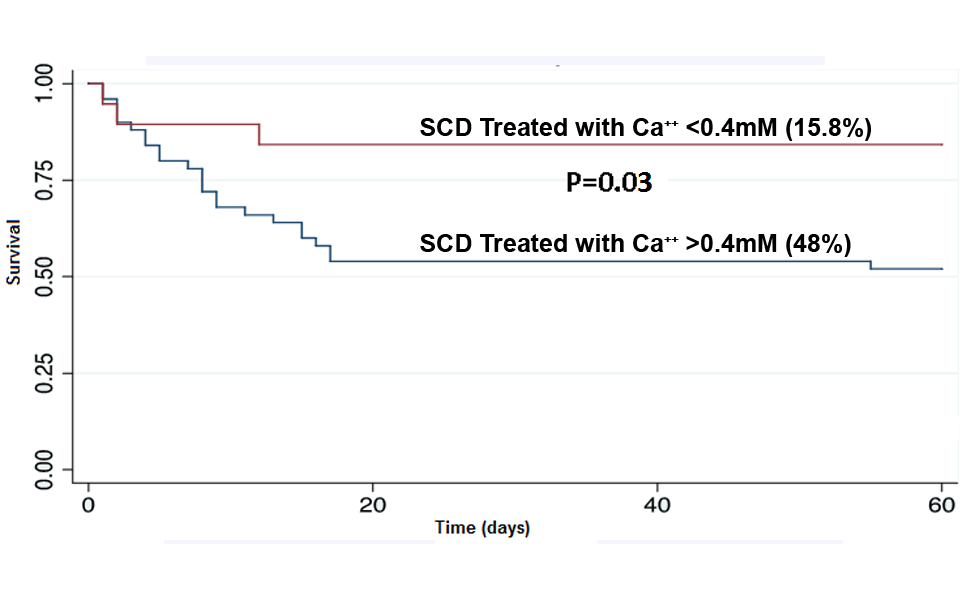

Supplement: S9 Fig — (TIF) [file pone.0132482.s010.tif]
