# Supplementary material for: A Multi-Center, Randomized, Controlled, Pivotal Study to Assess the Safety and Efficacy of a Selective Cytopheretic Device in Patients with Acute Kidney Injury
Source: PLoS One. 2015 Aug 5;10(8):e0132482. doi: 10.1371/journal.pone.0132482 (PMC4526678; doi:10.1371/journal.pone.0132482)
Supplement: S1 Protocol — (PDF) [file pone.0132482.s011.pdf]

A Multi-Center, Randomized, Controlled, Pivotal Study To Assess the Safety and Efficacy of A Selective Cytopheretic Device (SCD) In Patients with Acute Kidney Injury (AKI)

Protocol Number:	*SCD-003*	*IDE G090189*
Date:	*October 24th 2011*	*Version 1.4*
Sponsor:	*CytoPherx, Inc*<br>*401 West Morgan Road.*<br>*Ann Arbor, MI 48108*	

CytoPherx
Chief Medical Officer:

Alexander S. Yevzlin M.D.
401 West Morgan Road
Ann Arbor, MI 48108
Telephone:734.272.4772
Mobile: 608.658.9657
Email: ayevzlin@cytopherx.com

10/26/2011

Signature *Date*

CytoPherx
Director Clinical Affairs

J. Ricardo Da Silva RN BSN
401 West Morgan Road
Ann Arbor, MI 48108
Telephone:734.272.4772
Mobile: 917.763.4556
Email: rdasilva@cytopherx.com

10/26/ 2011

Signature *Date*

TABLE OF CONTENTS

1. INTRODUCTION 9
1.1. RATIONALE FOR THERAPY 9
2. PRIOR CLINICAL EXPERIENCE 10
2.1. PHASE II RENAL ASSIST DEVICE STUDY (RAD003 IND NUMBER 11077) 10
2.2. ESRD SAFETY AND BIOINFLAMMATORY ASSAY STUDY 12
2.3. AKI SAFETY, MORTALITY AND DEVICE INTEGRITY STUDY (CHINA) 13
2.3.1. STUDY DESIGN AND OBJECTIVE 13
2.3.2. CITRATE PROTOCOL 14
2.3.3. PATIENT POPULATION 14
2.3.4. STUDY ENDPOINTS 14
2.3.4.1. CLINICAL EFFICACY 14
2.3.4.2. PATIENT SAFETY 14
2.3.4.3. DEVICE INTEGRITY AND PERFORMANCE 14
2.3.5. STATISTICAL ANALYSIS 15
2.3.6. RESULTS 15
2.3.7. SUMMARY 17
2.4. PILOT STUDY USA ARF-002 - IDE G090189 17
2.4.1. NUMBER OF SUBJECTS ENROLLED 17
2.4.2. SAFETY 17
3. PIVOTAL STUDY (PROTOCOL SCD-003) OBJECTIVES AND ENDPOINTS 19
3.1. PRIMARY OBJECTIVE 19
3.2. SECONDARY OBJECTIVE 19
3.3. PRIMARY ENDPOINT 20
3.4. SECONDARY ENDPOINTS 20
4. STUDY DESIGN 20
5. SELECTION OF PATIENT POPULATION 20
5.1. PATIENT RECRUITMENT 20
5.2. INCLUSION CRITERIA 21
5.3. EXCLUSION CRITERIA 21
6. STUDY ACTIVITIES 22
6.1. SCREENING PERIOD 22
6.2. RANDOMIZATION 22
6.2.1. CONTROL ARM 23
6.2.2. STUDY TREATMENT ARM 23
6.3. BASELINE PERIOD 23

6.4. STUDY OBSERVATION PERIOD 24
6.5. STUDY FOLLOW UP PERIOD 26
6.5.1. POST END OF STUDY TREATMENT PERIOD 26
6.5.2. DAY 28 POST RANDOMIZATION 26
6.5.3. DAY 60 POST RANDOMIZATION 27
7. TREATMENT INTERRUPTIONS – DISCONTINUATION CRITERIA 27
7.1. TREATMENT INTERRUPTION CRITERIA 27
7.2. TREATMENT DISCONTINUATION CRITERIA 27
7.2.1. CLINICAL CRITERIA 27
7.2.2. NON-CLINICAL 28
8. CLINICAL TRIAL TERMINATION CRITERIA 29
9. SAFETY 29
9.1. ADVERSE EVENTS 29
9.2. SERIOUS ADVERSE EVENTS 30
9.3. UNANTICIPATED ADVERSE DEVICE EFFECT 30
9.4. ANTICIPATED ADVERSE EVENT 30
9.5. CAUSALITY 31
9.5.1. DEFINITELY RELATED 31
9.5.2. PROBABLY RELATED 31
9.5.3. POSSIBLY RELATED 31
9.5.4. UNRELATED 31
9.6. SEVERITY – INTENSITY 31
9.6.1. MILD 31
9.6.2. MODERATE 32
9.6.3. SEVERE 32
9.7. ADVERSE EVENT REPORTING 32
9.7.1. SCREENING PERIOD (CONSENT THROUGH RANDOMIZATION) 32
9.7.2. RANDOMIZATION THROUGH END OF STUDY 32
10. CLINICAL EVENTS COMMITTEE 33
11. DATA SAFETY MONITORING BOARD 33
12. STATISTICAL ANALYSIS 33
13. ECONOMIC AND QUALITY OF LIFE (QOL) EVALUATION 40
13.1. QOL AND ECONOMIC STUDY OBJECTIVES 40
14. DATA COLLECTION – STUDY MONITORING AND AUDIT 41
15. INVESTIGATOR’S STATEMENT OF RESPONSIBILITY 42
16. REFERENCES 43

PROTOCOL SYNOPSIS

**Study Title**	A Multi-Center, Randomized, Controlled, Pivotal Study To Assess the Safety and Efficacy of A Selective Cytopheretic Device (SCD) In Patients with Acute Kidney Injury (AKI)
**Study Phase**	Pivotal Study.
**Product Description**	The Selective Cytopheretic Device (SCD) is comprised of tubing, connectors, and a synthetic membrane cartridge. The device is connected in series to a commercially available Continuous Renal Replacement Therapy (CRRT) device. Blood from the CRRT circuit is diverted after the CRRT hemofilter through to the extra capillary space (ECS) of the SCD. Blood circulates through this space and it is returned to the patient via the venous return line of the CRRT circuit. Regional citrate anticoagulation is used for the entire CRRT and SCD blood circuits.<br><br>The SCD-ARF is a synthetic membrane with the ability to bind activated leukocytes and when used in a continuous renal replacement extracorporeal circuit in the presence of regional citrate anticoagulation modulates inflammation.
**Rationale**	Our primary hypothesis is that up to seven sequential 24-hour SCD treatments will improve survival in patients with Acute Kidney Injury (AKI) as compared to CRRT alone (standard of care). Further, SCD therapy may reduce the duration of maintenance dialysis secondary to AKI due to acute tubular necrosis (ATN). ATN is an acute reversible process and if not reversed in three months, it may not be reversed at all. [1, 2] Studies suggest that approximately 10% of AKI patients do not recover renal function [3, 4] and, therefore, require chronic dialysis.<br><br>Importantly, acute kidney injury is a highly lethal condition in critically ill patients. Despite improvements in acute medical care and advances in dialysis therapies, the mortality rate during the past four decades of this condition has not improved. Critically ill patients with AKI in hospital ICU settings have mortality rates of 50 to 80%. [5 - 9]<br><br>AKI promotes a systemic inflammatory response syndrome (SIRS) which results in systemic microvascular damage and, if severe, multi-organ dysfunction. [10, 11] Activated circulating leukocytes play a central role in this process. [12] Leukocytes, especially neutrophils, are major contributors to the pathogenesis and progression of many inflammatory

	disorders, including SIRS, sepsis, ischemia reperfusion injury, and acute respiratory distress syndrome (ARDS). Many therapeutic approaches are under investigation to limit the activation and tissue accumulation of leukocytes at sites of inflammation to minimize tissue destruction and disease progression. [13-15]
**Primary Objective**	To achieve a clinically and statistically significant improvement on all-cause mortality through Day 60 post randomization of CRRT + SCD treatment compared to CRRT alone.
**Secondary Objective**	To assess the effect of SCD treatment on various measures of patient clinical outcome and to evaluate the integrity of the SCD-ARF and patient safety in SCD treatments from the time of initiation of therapy to as many as seven consecutive 24-hour SCD treatments.
**Primary Endpoints**	The Primary Clinical Efficacy endpoint in this trial is time to all-cause mortality through 60 days post-randomization.
**Secondary Endpoints**	Renal Replacement Therapy dependency at day 60.<br>Mortality at day 28.<br>Number of ventilator free days (VFD) at day 28.<br>Mortality of Severe Septic patient sub-population at day 60.
**Study Populations**	Patients receiving care in the Intensive Care Units of each participating hospital will be screened daily and identified for participation in the trial.
**Study Design**	This is a two-arm, randomized, open-label, controlled multi-center Pivotal study.
**Approximate Number of Subjects**	Up to 344 patients will be enrolled in this study.
**Approximate Number of Centers**	Up to 30 Clinical Centers in the United States will participate in this study.
**Duration of Subject Participation**	Each patient will be followed for 60 days following randomization.
**Inclusion Criteria**	1. A patient, or legal representative, has signed a written informed consent form.<br>2. Must be receiving medical care in an intensive care unit (e.g., ICU, MICU, SICU, CTICU, Trauma).<br>3. Age 18 to 80 years.

	4. Females of child bearing potential who are not pregnant (confirmed by a negative serum pregnancy test) and not lactating if recently post-partum.<br>5. Must be receiving and tolerating CRRT therapy for a minimum of 4 hours, but not longer than 24.<br>6. Expected to remain in the ICU for at least 96 hours after evaluation for enrollment.<br>7. A clinical diagnosis of ATN due to hemodynamic or toxic etiologies. ATN is defined as Acute Kidney Injury occurring in a setting of acute ischemic or nephrotoxic injury with oliguria (average <20 mL/hr) for >6–12 hours ***or:*** an increase in serum creatinine ≥2 mg/dL (≥1.5 mg/dL in females) over a period of ≤4 days. (Note: Prerenal, hepatorenal, vascular, interstitial, glomerular, and obstructive etiologies are excluded on clinical or other diagnostic grounds.)<br>8. At least one non-renal organ failure (SOFA organ system score ≥2), as defined in Appendix A or presence (proven or suspected) of sepsis as defined in Appendix C.<br>9. All patients must be able to tolerate regional citrate anticoagulation.
**Exclusion Criteria**	1. Irreversible brain damage based on available historical and clinical information.<br>2. Presence of a renal transplant at any time.<br>3. Non-renal organ transplantation within six months of screening.<br>4. Presence of preexisting advanced chronic renal failure (Stage 5) prior to this episode of AKI. Preexisting Chronic Renal Failure is defined by dialysis dependence or as a baseline serum creatinine >2.5 mg/dL (men) or >2.0 mg/dL (women).<br>5. AKI occurring in the setting of burns, obstructive uropathy, allergic interstitial nephritis, acute or rapidly progressive glomerulonephritis, vasculitis, hemolytic-uremic syndrome, thrombotic thrombocytopenic purpura (TTP), malignant hypertension, scleroderma renal crisis, atheroembolism, functional or surgical nephrectomy, hepatorenal syndrome, cyclosporine or tacrolimus nephrotoxicity.<br>6. Metastatic malignancy which is actively being treated or may be treated by chemotherapy or radiation during the subsequent three month period after study therapy.<br>7. Chronic immunosuppression (e.g., HIV/AIDS, chronic glucocorticoid therapy >20 mg/day prednisone equivalent on a chronic basis). The acute use of glucocorticoids is permissible.<br>8. Severe liver failure as documented by a Child-Pugh Liver

	Failure Score >12 (see Appendix F).<br>9. Do Not Resuscitate (DNR) status.<br>10. Comfort measures only.<br>11. Patient is moribund or for whom full supportive care is not indicated.<br>12. Patient not expected to survive 28 days because of an irreversible medical condition. (This is not restrictive to AKI, and may include situations such as the presence of irreversible brain damage, untreatable malignancy, inoperable life threatening condition, or any condition to which therapy is regarded as futile by the PI.)<br>13. Any medical condition that the Investigator thinks may interfere with the study objectives.<br>14. Physician refusal.<br>15. Patient is a prisoner.<br>16. Dry weight of >150 kg.<br>17. More than one hemodialysis treatment during this hospital admission or prior to transfer from an outside hospital.<br>18. Platelet count <30,000/mm^3 at time of screening.<br>19. Concurrent enrollment in another interventional clinical trial. Patients enrolled in clinical trials where only measurements and/or samples are taken (NO TEST DEVICE OR TEST DRUG USED) are allowed to participate.<br>20. Use of any other Investigational drug or device within the previous 30 days.
**Evaluation Plan**	The primary endpoint in this trial is time to all-cause mortality through 60 days post-randomization. The primary analysis is the assessment of difference between the experimental treatment (CRRT+SCD) and the control treatment (CRRT alone) on the primary endpoint, and the assessment of this difference will be carried out at an overall two-sided 0.05 level of significance. Statistical treatment comparisons on secondary endpoints will also be carried out at an overall two-sided 0.05 level of significance, unless otherwise specified. Statistical analyses will be carried out using SAS version 9.1 or higher, and other appropriate statistical software if necessary.

LIST OF ABBREVIATIONS

AE	Adverse Event
AKI	Acute Kidney Injury
ARDS	Acute Respiratory Distress Syndrome
ARF	Acute Renal Failure
ATN	Acute Tubular Necrosis
Ca_i	Ionized Calcium
CBC	Complete Blood Count
CRF	Case Report Form
CRO	Clinical Research Organization
CRRT	Continuous Renal Replacement Therapy
CTICU	Cardiothoracic Intensive Care Unit
CVVH	Continuous Veno-Venous Hemofiltration
CVVHD	Continuous Veno-Venous Hemodialysis
CVVHDF	Continuous Veno-Venous Hemodiafiltration
ECS	Extra Capillary Space
ICU	Intensive Care Unit
HPTC	Human Proximal Tubular Cells
LOS	Length of Stay
MICU	Medical Intensive Care Unit
PEEP	Positive End Expiratory Pressure
QOL	Quality of Life
RAD	Renal Assist Device
SAE	Serious Adverse Event
SCD	Selective Cytopheretic Device
SICU	Surgical Intensive Care Unit
SIRS	Systemic Inflammatory Response Syndrome
SOFA	Sequential Organ Failure Assessment
WBC	White Blood Cell
UADE	Unanticipated Adverse Device Effect

1. INTRODUCTION

The Selective Cytopheretic Device (SCD) is comprised of tubing, connectors, and a synthetic membrane cartridge. The device is connected in series to a commercially available Continuous Renal Replacement Therapy (CRRT) device. Blood from the CRRT circuit is diverted after the CRRT hemofilter through to the extra capillary space (ECS) of the SCD. Blood circulates through this space and it is returned to the patient via the venous return line of the CRRT circuit. Regional citrate anticoagulation is used for the entire CRRT and SCD blood circuits.

The SCD-ARF is a synthetic membrane with the ability to bind activated leukocytes and when used in a continuous renal replacement extracorporeal circuit in the presence of regional citrate anticoagulation modulates inflammation.

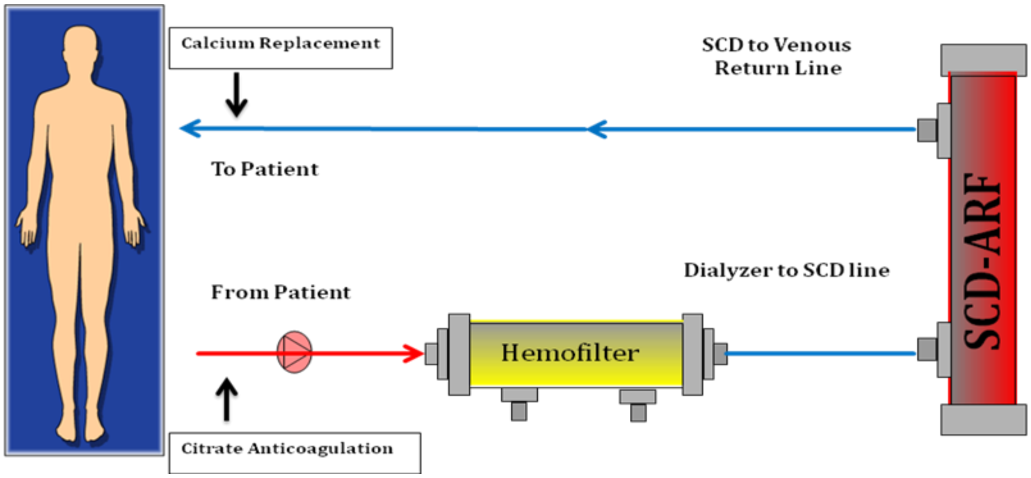

Figure 1 Schematic of the CRRT circuit and SCD

1.1. RATIONALE FOR THERAPY

Our primary hypothesis is that up to seven sequential 24-hour SCD treatments will improve survival in patients with Acute Kidney Injury (AKI) as compared to CRRT alone (standard of care). Further, SCD therapy may reduce the duration of maintenance dialysis secondary to AKI due to acute tubular necrosis (ATN). ATN is an acute reversible process and if not reversed in three months, it may not be reversed at all. [1, 2] Studies suggest that approximately 10% of AKI patients do not recover renal function [3, 4] and, therefore, require chronic dialysis.

Importantly, acute kidney injury is a highly lethal condition in critically ill patients. Despite improvements in acute medical care and advances in dialysis therapies, the mortality rate during the past four decades of this condition has not improved. Critically ill patients with AKI in hospital ICU settings have mortality rates of 50 to 80%. [5 - 9]

AKI promotes a systemic inflammatory response syndrome (SIRS) which results in systemic microvascular damage and, if severe, multi-organ dysfunction. [10,

11] Activated circulating leukocytes play a central role in this process. [12] Leukocytes, especially neutrophils, are major contributors to the pathogenesis and progression of many inflammatory disorders, including SIRS, sepsis, ischemia reperfusion injury, and acute respiratory distress syndrome (ARDS). Many therapeutic approaches are under investigation to limit the activation and tissue accumulation of leukocytes at sites of inflammation to minimize tissue destruction and disease progression. [13-15]

2. PRIOR CLINICAL EXPERIENCE

2.1. PHASE II RENAL ASSIST DEVICE STUDY (RAD003 IND NUMBER 11077)

CytoPherx predecessor, RenaMed, had implemented and participated in Phase I/II and Phase II clinical studies of renal cell therapy with the cellular-based Renal Assist Device (RAD) in ICU patients with AKI and multi-organ failure. The devices used in these studies were seeded with human renal proximal tubule cells isolated from kidneys donated for cadaveric transplantation, but found to be unsuitable for transplantation due to anatomic or fibrotic defects. The studies incorporated acellular cartridges (currently referred to as SCD) as a control and therefore provided supporting evidence for the safety of treatment with the SCD.

A Phase IIb clinical study of the RAD was undertaken to evaluate a commercial cell manufacturing process and the addition of citrate regional anticoagulation. Small subsets of patients were treated with citrate receiving either an acellular or a cell-containing cartridge. Subsets were analyzed to compare the mortality rates in patients treated with acellular cartridges (SCD) with systemic heparin anticoagulation or citrate regional anticoagulation.

Twenty-four patients were randomly assigned to the acellular cartridge (SCD) group that received either systemic heparin (n = 12) or regional citrate (n = 12) anticoagulation. As detailed in Table 1, baseline demographics of the subgroups were comparable with similar SOFA scores, organ-failure number, and incidence of sepsis between the two groups.

	Heparin (n=12)	Citrate (n=12)
Age (y)	61.4 ± 1.4	57.7 ± 5.3
Male/Female	10/2	7/5
SOFA	13.4 ± 1.1	12.2 ± 0.9
MOF	4.17 ± 0.46	3.33 ± 0.36
Sepsis	58%	58%

Notes: MOF = multi-organ failure number, SOFA = sequential organ failure assessment score. Organ failure defined as SOFA organ-specific score ≥ 2

Table 1 RAD003 Baseline Subgroup Demographics

Figure 2 displays the survival plot for the two subgroups. The mortality rate in the heparin patient group was 50% vs. 25% in the citrate-treated group (n = 12 for each treatment arm) at 28 days and 75% vs. 33%, respectively, at 90 days (Chi-square < 0.05).

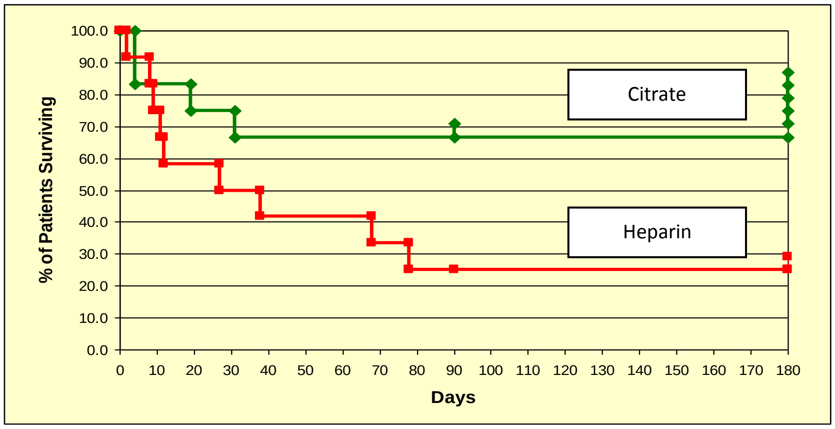

Figure 2 RAD003 Survival plot

Table 2 summarizes the reasons for discontinuation of the continuous venovenous hemofiltration (CVVH) + acellular cartridge (SCD) in the AKI patients along with the duration of therapy on the device. The median duration of treatment on the device was 62 hours.

Failure of CVVH circuit	30%
Completed 72 hrs of therapy	26%
Failure of acellular device	17%
Improvement in clinical status	9%
Concomitant medical conditions	9%
Withdrawal of participation	4%
Device-related medical events	4%
Death	0%
Notes: Median time of therapy = 2.6 d (62 h)	

Table 2 Reasons for discontinuation of CVVH+Acellular Cartridge (currently refer to as SCD) therapy (n=24)

Device-related medical events resulted in discontinuation of one patient assigned to receive the SCD (4%). The reason for discontinuation of the CVVH + acellular cartridge (SCD) in the heparin group was a low platelet count. Thrombocytopenia is a recognized complication of critically ill patients in the ICU. Review of the total white blood cell, neutrophil, and platelet counts obtained daily during the treatment period did not show any substantive change from baseline values in either of the two groups. Except for the one patient noted above, none of the patients developed absolute neutropenia or thrombocytopenia during the treatment period.

For patients listed as having "Failure of the acellular Device," regardless of treatment group, the case report form (CRF) indicated that this was due to clotting of the CVVH circuit. Thus, in each case, clotting originated in the CVVH circuit and then propagated to the cellular or acellular circuit cartridge. Combining the

"Failure of the CVVH Circuit" and "Failure of the Acellular Device" categories the incidence of clotting was 47% in the acellular control group before the complete 72-hour treatment plan, with median time of blood flow patency of the circuit of 62 hours. Compared to single-cartridge continuous renal replacement therapy (CRRT), this time-to-failure rate is favorable. [16-19]

Immuno-fluorescent staining of a small number of SCD cartridges after patient treatment demonstrated adherent leukocytes on the outer surface of the hemofilter membranes. Elution of cells from the SCD's ECS demonstrated that >98% of the cells were neutrophils.

All serious adverse events (SAEs) during the first 28 days after randomization were reported to the Medical Monitor within 24 hours of the event. The events were initially reported in a blinded manner, but after study completion, the blind was broken and the SAEs were analyzed by treatment group. SAEs were reported in 57.1% (16 of 28) patients receiving CVVH + RAD and in 52.0% (13 of 25 patients) receiving CVVH + acellular cartridge (SCD).

The reported SAEs were consistent with a seriously ill ICU patient population with AKI receiving CVVH. When analyzed by body system, a higher frequency of serious cardiac-related adverse events was observed in the RAD treatment group (7 of 28; 25%) compared to the control acellular treatment group (2 of 25; 8%). The SAEs included cardiac arrest (10.7% and 4.0%, respectively), cardiopulmonary arrest (10.7% and 0%, respectively), cardiopulmonary failure (0% and 4.0%, respectively), and electromechanical dissociation (3.6% and 0%, respectively).

2.2. ESRD SAFETY AND BIOINFLAMMATORY ASSAY STUDY

A study of the SCD was conducted at the Henry Ford Hospital in Detroit, Michigan entitled "A Phase I/II Trial of a Two-Cartridge Hemodialysis System on Inflammatory Markers in Chronic Hemodialysis Subjects". This study was designed to determine what effect one treatment with the SCD would have on the reduction of bioinflammatory markers such as cytokines IL-2, IL-6, IL-8 and IL-10 and white cell activation in chronically inflamed End Stage Renal Disease (ESRD). The study enrolled fifteen subjects at one site, with four of the original 15 patients re-enrolled in what was called "Amendment 2".

Fifteen chronic hemodialysis (HD) patients with elevated CRP levels were treated for four hours under standard HD with systemic heparin anticoagulation and, at their next dialysis treatment session, received standard HD plus SCD therapy with regional citrate anticoagulation. The 15 patients have completed the study treatment period and safety data is shown in Table 3 as follows:

Adverse Event	Number of Mild Severity Events	Number of Moderate Severity Events
Fever	1	0
Chills	2	0
Headache	1	0
Nausea	3	0
Vomiting	2	1
Diarrhea	1	0
Dizziness	1	1
Visual Disturbances	0	1
Lethargy	0	1
Itching	1	0
Latent TB infection	1	0
Cough	1	0
Left cheek swelling	1	0
Decreased hemoglobin	1	1
Increased CRP level	2	0
Neck swelling cellulites	0	1
Ankle sprain	0	1
Replacement of left dysfunctional IJ catheter for HD	1	0
Dialysis catheter fell off during sleep	0	1
Muscle cramp	0	1
Chest pain	0	1
Clotted extracorporeal system. 350 ml blood loss	0	1
L Upper quadrant pain	0	1
Decreased WBC	0	1

Table 3 Report of Adverse Events - ESRD trial

Additional data analysis and the assay of various biomarkers remain in progress.

2.3. AKI SAFETY, MORTALITY AND DEVICE INTEGRITY STUDY (CHINA)

2.3.1. STUDY DESIGN AND OBJECTIVE

A study of the SCD was conducted at the Huashan Hospital in Shanghai, China entitled “An Exploratory Clinical Study to Assess Safety and Efficacy of the Double Hemofiltration Cartridge Device (DCD) in Patients with Acute Renal Failure”.

This study was a prospective, non-randomized, interventional study designed to evaluate the effect of treatment with the SCD on in-hospital mortality in the acute renal failure population being treated with continuous veno-venous hemofiltration (CVVH) with regional citrate anticoagulation. All subjects received standard intensive care treatment for patients undergoing CVVH in

addition to the SCD treatment. CVVH treatment was delivered via standard modes, i.e. 2.5L/hour replacement fluid dose pre-dilution.

2.3.2. CITRATE PROTOCOL

The hypotonic trisodium citrate (4%, 136 mmol/L) was infused pre-filter at a fixed ratio of 3.5-4.5 mmol/L to the blood flow by means of a three-way stopcock at the junction of the catheter. The calcium-free replacement fluid delivered at the rate of 2.5L/h was composed of 121 mmol/L sodium, 3.2 mmol/L potassium, 0.75mmol/L magnesium, 109 mmol/L chloride, 7 mmol/L bicarbonate and 250mg/dL of dextrose. The calcium chloride solution (5%, 340mmol/L calcium) was infused into the venous return by means of a three-way stopcock at the junction of the catheter.

Calcium chloride was replaced at 1.0 mmol/L of blood flow as an initial dose. Systemic ionized calcium was checked repeatedly, and accordingly, calcium infusion rate was adjusted to maintain the level of calcium in circuit between 0.2-0.4 mmol/L and ionized systemic calcium at normal range (1.0-1.2 mmol/L).

2.3.3. PATIENT POPULATION

Adult male and female (non-pregnant) patients, aged 18-80 years, requiring either CVVH or CVVHD for the treatment of acute kidney injury secondary to acute tubular necrosis (ATN) were enrolled. ATN was defined as acute kidney injury occurring in a setting of acute ischemic or nephrotoxic injury and oliguria (<20 ml/hr) for > 24 hours or an increase in serum creatinine > 2 mg/dL (> 1.5 mg/dL in females) over a period of < 4 days.

2.3.4. STUDY ENDPOINTS

In this exploratory clinical study three domains of interest were designated, and within each of those domains both Key Endpoints and Exploratory Endpoints were pre-specified as noted below:

2.3.4.1. CLINICAL EFFICACY

The primary clinical efficacy endpoint was in-hospital all-cause mortality. In addition, urine output was assessed as a surrogate marker for renal recovery. Both of these clinical parameters were compared to data from historical controls from the PICARD dataset matched for age and SOFA score [20].

2.3.4.2. PATIENT SAFETY

The domain of patient safety and tolerability was assessed based upon the occurrence of adverse events (all, related, unexpected serious, unexpected serious related) as well as with leukocyte and platelet levels.

2.3.4.3. DEVICE INTEGRITY AND PERFORMANCE

Several parameters of device integrity and performance were monitored including evidence of leakage, cracking, clotting and hemolysis.

2.3.5. STATISTICAL ANALYSIS

In-hospital mortality data was compared between the SCD treated patients and the case-matched historical control using the Fisher exact test. Age and SOFA scores were compared between the latter groups using the paired T-test. Multiple regression analysis was performed with mortality as the dependant variable and the following independent variables in the regression equation: age, SOFA score, and average change in urine output over the first seven days during or after treatment, and treatment modality (SCD vs. CVVH historical cohort). Urine output and change in urine output between the SCD and controls were compared using ANOVA analysis. All other variables reported were analyzed using descriptive statistics only.

2.3.6. RESULTS

A total of 12 patients were enrolled in this study. Safety for this patient population is reported in Table 4. There were no SAEs reported.

Adverse Event (n=12 patients)	Number of Mild Severity Events	Number of Moderate Severity Events
Hypercalcemia	9	1
Thrombocytopenia	1	0
Hypocalcemia	0	0
Allergic Reaction	1	0
Hypophosphatemia	2	0
Hypernatrimia	1	0

Table 4 Adverse Events Reported for all 12 patients (including 3 off protocol)

Of the 12 patients enrolled in the study, three were enrolled outside of the inclusion/exclusion criteria. Two of these patients were not expected to survive for 96 hours and the third had active cancer. Therefore, the analysis focuses on the remaining 9 patients and is described below. [21]

The nine patients enrolled in the trial "on protocol" did not differ from the case matched controls with respect to age and SOFA score (Table 5). The mortality for the case matched controls was 77.78%, while the observed mortality in the SCD treatment group was 22.22% ($p = .027$). Multiple regression analysis identified treatment with SCD as the only significant variable affecting mortality among age, SOFA score, and average change in urine output over the first seven days during or after treatment, and treatment modality (SCD vs. CVVH historical cohort, ß = -0.5728; $p = 0.0222$).

	SCD		Matched Controls from PICARD			
	n=9		n=9			
	Mean	Std Dev	Mean	Std Dev	Test	p value
Age	59.33	13.85	59.22	17.15	T-test	0.99
SOFA	11	3.64	11.44	3.05	T-test	0.78
Mortality	22.22%		77.78%		Fisher's exact	0.027*

Table 5 SCD mortality and demographics compared to PICARD case-matched controls

Mean total urine output in the 9 subjects receiving SCD treatment increased from a baseline of approximately 500 ml/day to over 1,500/day by day 7 of treatment. While there was no statistically significant difference in the change in urine output over the treatment time between the SCD and control group, mean urine output increased over time in the SCD treated group and diminished over time in the PICARD case matched control group.

In the 9 subjects analyzed on SCD treatment, no neutropenic events were reported. Mean WBC counts remained normal throughout treatment, with a mild decline noted upon initiation of therapy that was shown to rebound by day 7.

No bleeding events were reported. Average platelet counts remained in the functional range (above 50,000) throughout treatment, with a mild decline noted upon initiation of therapy that was shown to plateau by day 4 to an average platelet count of 75,000.

Adverse events noted included hypercalcemia (8), hypocalcemia (1), hypophosphatemia (2), hypernatremia (1), and thrombocytopenia (1), with the last patient requiring a platelet transfusion upon a decline in platelet count below 20,000 (Table 6). These events were not attributed to the device by the investigator, as they commonly occur with CRRT treatment. Moreover, the thrombocytopenic AE was not reported as serious by the PI despite the necessity of platelet transfusion because there were no clinical sequelae of the thrombocytopenia (e.g. no bleeding, bruising).

Adverse Event (n=9 patients)	Number of Mild Severity Events	Number of Moderate Severity Events
Hypercalcemia	6	2
Thrombocytopenia	1	0
Hypocalcemia	0	1
Allergic Reaction	1	0
Hypophosphatemia	2	0
Hypernatremia	1	0

Table 6 Adverse event reporting – China Study – n=9 patients

2.3.7. SUMMARY

This non-randomized, single center pilot study evaluated the safety and efficacy of treatment with a selective cytopheretic device in patients with a conventional CRRT circuit. SCD treatment significantly reduced all-cause, in-hospital mortality in ICU patients with AKI compared to case matched controls from a national dataset (PICARD).

This improved survival was demonstrated to be independent of age and SOFA score. Treatment with the SCD was well tolerated with an adverse event profile expected for a seriously ill population in the ICU with AKI. The blood flow patency of the SCD was comparable to single-cartridge CRRT modalities.

2.4. PILOT STUDY USA ARF-002 - IDE G090189

The pilot study of the SCD device (ARF-002 Clinical Trial) was designed to evaluate the safety and effect of the SCD treatment after up to seven consecutive 24 hour SCD treatments compared to historic data on in-hospital mortality and all-cause mortality at day 28, and day 60 in the acute renal failure population being treated with Continuous Renal Replacement Therapy (CVVH, CVVHD, CVVHDF) with regional citrate anticoagulation.
The study was conducted in 6 Clinical Centers in the United States. Patients receiving care in the critical care units of each participating hospital were screened daily and identified for recruitment.

2.4.1. NUMBER OF SUBJECTS ENROLLED

A total of 35 subjects were enrolled by a total of 6 centers in the United States between May 2010 and January 2011.

2.4.2. SAFETY

A summary of all adverse events (AEs) observed in the 35 subjects are summarized in Table 7. A total of 199 adverse events were observed in 33 of the 35 subjects. Of these 199 AEs, 12 were deemed to be possibly related and 1 deemed definitely related (as determined by the investigator) to the study therapy. These included worsening coagulation defect, hypotension, neutropenia, DIC, thrombocytopenia, recurrent renal failure, hypophosphatemia, hypercalcemia, anemia, and cardiogenic shock. (Thrombocytopenia is frequently encountered during administration of CRRT therapy alone [22] and neutropenia is a known consequence of critical illness and/or sepsis [23]).

One of these 13 related events, one event was deemed to be “definitely” related to therapy which was coded as “Metabolic Alkalosis Secondary to Citrate.” Although this was the name given to the event by the PI, the event in question was a case of severe hypocalcemia which resulted from the initiation of citrate anticoagulation and which resulted in termination of therapy 2 hours after initiation. Citrate intolerance is a known side effect of citrate anticoagulation in the setting of liver disease. In some patients, citrate quickly builds up and leads

to metabolic alkalosis and hypocalcemia [24]. In this subject, the citrate intolerance was quickly recognized and therapy was discontinued 2 hours after initiation.

Two subjects experienced thrombocytopenia which was deemed possibly related to the therapy and one required platelet transfusions. There were 8 additional incidences of thrombocytopenia in 8 subjects that were deemed as unrelated by the PI. Four of these 8 subjects required platelet transfusions. As stated above, thrombocytopenia is frequently encountered during administration of CRRT therapy alone [22] and is often a consequence of the types of critical illnesses (e.g., bone marrow suppression, SIRS, sepsis, drug reactions) observed in the ICU setting. For example, in a retrospective analysis of a 71 patient cohort in the ICU on systemic heparin anticoagulation who received CRRT, 31 patients (44%) experienced severe thrombocytopenia [22].

Of the 199 events, 34.7% were deemed to be mild and were experienced by 60% of the subjects, 51.8% were moderate and experienced by 71% of the subjects and 13.6% were severe, experienced by 54% of the subjects. The AEs observed were those that were expected for a critically ill patient population with acute renal failure and/or in an ICU setting.

Adverse Events	Number of Subjects with Events N (% of 35)	Number of Events % of 199 (n/199)
**Total adverse events**	33 (94%)	199
**Serious adverse event**	23 (66%)	14.1% (28/199)
**Unanticipated Adverse Device Effect**	0 (0%)	0.0% (0/199)
**Relationship to Study***		
Unrelated to study therapy	32 (91%)	93.5% (186/199)
Possibly related to study therapy	8 (23%)	6.0% (12/199)
Probably related to study therapy	0 (0%)	0.0% (0/199)
Definitely related to study therapy	1 (3%)	0.5% (1/199)
**Outcome**		
Resolved w/ sequelae	14 (40%)	13.6% (27/199)
Resolved w/out sequelae	25 (71%)	64.3% (128/199)
Continuing	11 (31%)	11.1% (22/199)
Death	11 (31%)	5.5% (11/199)
Reported as Unknown	3 (9%)	5.5% (11/199)
**Frequency**		
Single Episode	29 (83%)	54.3% (108/199)
Intermittent	16 (46%)	21.1% (42/199)
Continuous	16 (46%)	24.1% (48/199)
Reported as Unknown	1 (3%)	0.5% (1/199)
**Severity**		
Mild	21 (60%)	34.7% (69/199)
Moderate	25 (71%)	51.8% (103/199)
Severe	19 (54%)	13.6% (27/199)

*Subjects experienced more than one AE, therefore, numbers do not add up to N=22 (number of subjects experiencing any adverse event).

Table 7 Summary of Adverse Events ARF-002

Clinical outcomes for the 35 subjects enrolled are shown in Table 8. Death from any cause at Day 60 was 31.4%. This compares very favorably with the greater than 50% mortality reported in the literature [25] for subjects with similar SOFA scores.

Renal recovery was observed in all of the surviving subjects at Day 60. The mean time to ICU discharge from the beginning of SCD therapy was 15.0±9.1 days [CI 11.1,18.9] and the time to hospital discharge from the beginning of SCD therapy was 21.8±10.9 days [CI 17.0,26.6].

Clinical Efficacy	Outcome (N=35 Subjects)†	95% CI
**Primary Endpoint**		
Death in hospital	28.6% (10/35)	[14.6%,46.3%]
Death from any cause by day 28 from time of enrollment	25.7% (9/35)	[12.5%,43.3%]
Death from any cause by day 60 from time of enrollment	31.4% (11/35)	[16.9%,49.3%]
**Other Clinical Efficacy**		
Time to ICU discharge (days) from enrollment	15.0±9.1 (23)	[11.1,18.9]
Time to hospital discharge (days) from enrollment	21.8±10.9 (22)	[17.0,26.6]
No. of subjects discharged from ICU by day 28	60.0% (21/35)	[42.1%,76.1%]
No. of subjects discharged from hospital by day 28	54.3% (19/35)	[36.6%,71.2%]
No. of subjects discharged from ICU by day 60	65.7% (23/35)	[47.8%,80.9%]
No. of subjects discharged from hospital by day 60	62.9% (22/35)	[44.9%,78.5%]
Renal Recovery to day 28*	73.1% (19/26)	[52.2%,88.4%]
Renal Recovery to day 60*	100% (24/24)	[86.2%.100%)

† The presented numbers refer to: for discrete variable, % (no. patients with events/ total no. available patients) for continuous variable, Mean±SD(N), where N is the number of available patients.
* Does not include subjects who died (N=5 at day 28 and N=7 at day 60)

Table 8 Clinical Efficacy Outcome ARF-002

All safety data has been collected by the Clinical Research Organization (CRO), SAE and death notices are sent directly to CRO from the Principal Investigator with supporting source documentation. The CRO safety officer reviews this information, makes queries as necessary, and generates a narrative of the events for monthly safety reports that are sent to CytoPherx.

3. PIVOTAL STUDY (PROTOCOL SCD-003) OBJECTIVES AND ENDPOINTS

3.1. PRIMARY OBJECTIVE

To achieve a clinically and statistically significant improvement on all-cause mortality through day 60 post randomization of CRRT + SCD treatment compared to CRRT alone

3.2. SECONDARY OBJECTIVE

To assess the effect of SCD treatment on various measures of patient clinical outcome, health economics and to evaluate the integrity of the SCD-ARF and patient safety in SCD treatments from the time of initiation of therapy to as many as seven consecutive 24-hour SCD treatments.

3.3. PRIMARY ENDPOINT

The Primary Clinical Efficacy endpoint measure in this trial is time to all cause mortality through 60 day post randomization.

3.4. SECONDARY ENDPOINTS

- Renal Replacement Therapy dependency at day 60.
- Mortality at day 28.
- Number of ventilator free days (VFD) at day 28.
- Mortality of Severe Septic patient sub-population at day 60.

Secondary Endpoints are defined in Appendix D.

4. STUDY DESIGN

This is a two-arm, randomized, open-label, controlled multi-center Pivotal study.

5. SELECTION OF PATIENT POPULATION

5.1. PATIENT RECRUITMENT

Up to 30 Clinical Centers in the United States will enroll up to 344 patients to participate in this clinical trial. Patients receiving care in the Intensive Care Units of each participating hospital will be screened daily and identified for participation in the trial.

All patients that are screened for the study will be entered by the Study Staff on a screening log. If the patient is not enrolled, the screening log will include information explaining why enrollment did not take place (exclusion criteria, attending physician refusal, patient refusal, etc).

All enrolled patients will receive standard intensive care treatment for patients undergoing CRRT or CRRT + SCD. Any mode of CRRT will be allowed (CVVH, CVVHD, and CVVHDF) following each participating clinical site's protocol. The modality of CRRT is not expected to affect performance of the SCD.

The Sponsor will provide each site with a CRRT device (B.Braun Diapact® CRRT System) to be used with each patient randomized either to the Control or Study Arms. The B.Braun® CRRT System is a compact, self-containing system for high-flow continuous or intermittent renal therapies. Some of the technical features of the Diapact include an integrated plate warmer for the heating of infusion and dialysate fluids and a single load cell weighing system which reduces the possibility of error and increases accuracy. The Sponsor will also provide all the tubing, SCD-ARF and hemofilters to be used during the observation period of this trial.

Each participating clinical site is to use their regional citrate anticoagulation protocol for the CRRT and SCD-ARF circuits (Study Arm) and for the CRRT only (Control Arm). The recommended ionized calcium level (measured post

SCD-ARF) in the CRRT and SCD-ARF blood circuit should be between 0.25 and 0.4 mmol/L.

5.2. INCLUSION CRITERIA

1. A patient, or legal representative, has signed a written informed consent form.
2. Must be receiving medical care in an intensive care unit (e.g., ICU, MICU, SICU, CTICU, Trauma).
3. Age 18 to 80 years.
4. Females of child bearing potential who are not pregnant (confirmed by a negative serum pregnancy test) and not lactating if recently post-partum.
5. Must be receiving and tolerating CRRT for a minimum of 4 hours, but not longer than 24 hours.
6. Expected to remain in the ICU for at least 96 hours after evaluation for enrollment.
7. A clinical diagnosis of ATN due to hemodynamic or toxic etiologies. ATN is defined as Acute Kidney Injury occurring in a setting of acute ischemic or nephrotoxic injury with oliguria (average <20 mL/hr) for >6–12 hours ***or:*** an increase in serum creatinine ≥2 mg/dL (≥1.5 mg/dL in females) over a period of ≤4 days. (Note: Prerenal, hepatorenal, vascular, interstitial, glomerular, and obstructive etiologies are excluded on clinical or other diagnostic grounds.)
8. At least one non-renal organ failure (SOFA organ system score ≥2), as defined in Appendix A or presence (proven or suspected) of sepsis as defined in Appendix C.
9. All patients must be able to tolerate regional citrate anticoagulation.

5.3. EXCLUSION CRITERIA

1. Irreversible brain damage based on available historical and clinical information.
2. Presence of a renal transplant at any time.
3. Non-renal organ transplantation within six month of screening.
4. Presence of preexisting advanced chronic renal failure (Stage 5) prior to this episode of AKI. Preexisting Chronic Renal Failure is defined as a baseline serum creatinine >2.5 mg/dL (men) or >2.0 mg/dL (women).
5. AKI occurring in the setting of burns, obstructive uropathy, allergic interstitial nephritis, acute or rapidly progressive glomerulonephritis, vasculitis, hemolytic-uremic syndrome, thrombotic thrombocytopenic purpura (TTP), malignant hypertension, scleroderma renal crisis, atheroembolism, functional or surgical nephrectomy, hepatorenal syndrome, cyclosporine or tacrolimus nephrotoxicity.
6. Metastatic malignancy which is actively being treated or may be treated by chemotherapy or radiation during the subsequent three month period after study therapy.
7. Chronic immunosuppression (e.g., HIV/AIDS, chronic glucocorticoid therapy >20 mg/day prednisone equivalent on a chronic basis). The acute use of glucocorticoids is permissible.
8. Severe liver failure as documented by a Child-Pugh Liver Failure Score >12 (see Appendix F).
9. Do Not Resuscitate Status (DNR).

10. Comfort measures only.
11. Patient is moribund or for whom full supportive care in not indicated.
12. Patient is not expected to survive 28 days because of an irreversible medical condition. (This is not restrictive to AKI, and may include situations such as the presence of irreversible brain damage, untreatable malignancy, inoperable life threatening condition, or any condition to which therapy is regarded as futile by the PI.)
13. Any medical condition that the Investigator thinks may interfere with the study objectives.
14. Physician refusal.
15. Patient is a prisoner.
16. Dry weight >150 kg.
17. More than one hemodialysis treatment during this hospital admission or prior to transfer from an outside hospital.
18. Platelet count <30,000/mm^3.
19. Concurrent enrollment in another interventional clinical trial. Patients enrolled in clinical trials where only measurements and or samples are taken (NO TEST DEVICE OR TEST DRUG USED) are allowed to participate.
20. Use of any other interventional drug or device within the previous 30 days.

6. STUDY ACTIVITIES

6.1. SCREENING PERIOD

The screening period is the time period when a patient is identified, informed consent is obtained, and the patient is evaluated for inclusion in the clinical trial. Because of the critically ill nature of the study population, it is expected that the majority of patients will not be able to provide informed consent. Where subjects are unable to consent themselves, surrogate informed consent will be sought from the subject’s healthcare proxy or other equivalent legal representative. Only adverse events directly related to the screening procedures will be reported during this time.

Medical history and test results must be analyzed to ensure the patient meets all eligibility criteria. Tests that are specific to this protocol (i.e. not standard of care) require the patient or legal representative to have provided written consent for participation.

Any patients that do not meet study criteria, whether consent was obtained or not, will not be randomized and enrolled into the study. These patients will not be counted in the overall study enrollment numbers, but will be listed on the screening log.

6.2. RANDOMIZATION

A centralized randomization system has been established with 24-hour availability to provide a mechanism to randomize subjects and enroll them in the study. Once the patient meets all eligibility criteria, **including being on CRRT**

for a minimum of 4 hours, but no longer than 24, and has signed informed consent, the site will enter the patient into the randomization system.

Patients will be randomized in a 1:1 allocation utilizing a random permuted block design into either the control or treatment arm, stratified by study center and the presence of severe sepsis or not. (Appendix C). This should occur as close to the start of the Observation period as possible while also allowing time for baseline tests and data to be completed before the patient has been on CRRT for 24 hours. Patients will then be considered enrolled in the study and given a study number. Randomization results will not be revealed to the clinical team and patient until immediately prior to start of first treatment.

6.2.1. CONTROL ARM

Patients randomized to the control arm will receive renal replacement therapy utilizing a CRRT device that is identical to that used for the SCD treatment, tubing and hemofilter provided by the Sponsor. Anticoagulation of the system will be done using the institution's protocol for citrate anticoagulation. All patients in the control arm are required to use citrate as an anticoagulant.

6.2.2. STUDY TREATMENT ARM

Patients randomized to the study treatment arm will receive renal replacement therapy plus SCD using the CRRT device, first hemofilter, SCD-ARF, and all tubing provided by the sponsor. Anticoagulation of the CRRT and SCD system will be done using the institution's protocol for citrate anticoagulation. All patients in the treatment arm are required to use citrate as anticoagulant.

6.3. BASELINE PERIOD

The Baseline Period begins at randomization and ends at the start of the Observation Period. The following information will be recorded as close to the start of the Observation Period as possible. Data from standard of care tests may be used as baseline data, as long as it is captured within the time frames noted.

- **Demographic Data**
  - Date of birth
  - Gender
  - Race/ethnicity
- **Hospitalization Data**
  - Hospital Admission Date
  - Hospital Admission Diagnosis
  - ICU Admission Date
  - ICU Admission Diagnosis
- **Vital Signs/Physical Assessment**
  - Temperature
  - Blood Pressure, heart rate, body weight
  - EKG **(within 12 hours prior to the start of Observation Period)**
  - Sequential Organ Failure Assessment (SOFA Score)

  - Physical Exam
  - Urine Output (previous 24 hours)
- **Clinical Laboratory Tests (within 12 hours prior to the start of Observation Period)**
  - CBC with differential
  - BUN/Creatinine
  - Na, K, Cl, HCO_3, Ca, Mg, Ionized Ca, PO_4
  - ALT, AST, LDH, Bilirubin, ALP, Total Protein, Albumin, Glucose
  - PT, PTT, INR
  - Haptoglobin
  - Urinalysis (if urine available)
- **Respiratory**
  - Status – Ventilator support (yes/no; date of intubation)
  - If on Vent: Mode, NO, FiO_2, Settings (TV, PEEP, RR)
  - If on Vent with an arterial line: Arterial Blood Gas (12 hours prior to start of Observation Period)
  - If on Vent without an arterial line: Arterial Blood Gas (24 hours prior to start of Observation Period)
  - If not Vented: Supplemental oxygen requirements
- **Blood for Research**
  - Blood sample will be sent to an outside laboratory for testing of biomarkers such as IL6, IL10, Elastase, and GCSF.
- **Other (Event-driven)**
  - All the medications and blood products administered to the patient in the 24 hours prior to prior to start of Observation Period.
  - Adverse Events occurring since randomization, unless directly related to screening procedure
  - Diagnostic/Therapeutic Procedures in the 24 hours prior to start of Observation Period.
  - Microbiology/culture data

6.4. STUDY OBSERVATION PERIOD

The Study Observation Period begins when the patient starts Control or Study Therapy and will continue up to a maximum of 168 hours from the time of Observation Start. It is expected that the patient will begin the Observation Period before the patient has reached 24 hours on CRRT.

This observation period has been broken into seven 24 hour time periods. It ends when the patient has reached 168 hours in the observation period or when the patient meets the termination criteria outlined in section 7.2 as determined by the Principal Investigator or Medical Team at the participating Clinical Site.

All tests are scheduled on a 24 hour cycle, or once a day, and can be scheduled to be drawn around each participating site's ICU blood drawing schedule, with the exception of any test that requires a specific draw frequency.

The following information will be obtained for patients enrolled in both study arms and recorded during the study observation period on a daily basis unless otherwise specified:

- **Vital Signs/Physical Assessment**
    - Temperature
    - Blood Pressure, heart rate – every 4 hours (+/- 1 hour)
    - Body weight
    - Sequential Organ Failure Assessment (SOFA Score)
    - Urine Output (previous 24 hours)
    - Net Fluid Balance (previous 24 hours)
- **Clinical Laboratory Tests**
    - CBC with differential – every 8 hours (+/- 2 hours)
    - BUN, Creatinine
    - Na, K, Cl, HCO_3, Ca, Mg, Ionized Ca,PO_4
    - ALT, AST, LDH, Bilirubin, ALP, Total Protein, Albumin, Glucose
    - Haptoglobin (on the last day of therapy only)
    - PT, PTT, INR
- **Respiratory**
    - Status – Ventilator support (yes/no; date of extubation)
    - If on Vent: Mode, FiO_2, Settings- TV, PEEP, RR (q4h first 12 hrs (+/- 30 min) of observation period and then q12h (+/- 1 hour) thereafter)
    - If on Vent with an arterial line: Arterial Blood Gas (q4h first 12 hrs (+/- 30 min) and then q12h (+/- 1 hour) thereafter)
    - If on Vent without an arterial line: Arterial Blood Gas (once per 24 hours)
    - If not Vented: Supplemental oxygen requirements (q4h first 12 hrs (+/- 30 min) of observation period and then q12h (+/- 1 hour) thereafter)
- **Blood for Research**
    - Collect at hours 24, 72, 120, and 168 (+/- 4 hours). If study observation period ends prior to hour 168, collect at end of study observation period. Blood for biomarkers will be collected as close as possible to the time of SCD scheduled changes in the days required (+/- 4 hours) if practical.
    - Blood sample will be sent to an outside laboratory for testing of biomarkers such as IL6, IL10, Elastase, and GCSF.
- **CRRT Parameters (Both study arms – start at hour 0)**
    - Parameters include modality, blood flow rate, net fluid removal, dialysate or replacement solution flow rate, citrate infusion rate, calcium replacement rate (every 6 hours +/- 1 hour)
    - System performance (circuit survival - event driven)
    - Post filter ionized calcium (every 6 hours +/- 1 hour)
- **SCD Performance (Treatment Arm Only – start at hour 0)**
    - SCD-ARF performance (event driven)
- **Other (Event Driven)**
    - All the medications and blood products administered to the patient.

- Adverse Events
- Diagnostic/Therapeutic Procedures
- Microbiology/culture data

6.5. STUDY FOLLOW UP PERIOD

6.5.1. POST END OF STUDY TREATMENT PERIOD

This Study Follow Up period has been broken into seven 24 hour time periods. It starts when the patient has reached 168 hours in the observation period or when the patient meets the termination criteria outlined in section 7.2. It ends at 168 hours or until ICU discharge, whichever comes first. (Except for SOFA, net fluid balance and pressor use, which are followed until ICU discharge or Day 28, whichever comes first and microbiology/culture data which will be collected until hospital discharge or Day 60, whichever comes first.)

Record the following data daily in the Study Follow Up Period unless otherwise noted:

- **Vital Signs/Physical Assessment**
  - Blood pressure and heart rate
  - SOFA Score (record until ICU discharge or Day 28)
  - Physical Exam (first 24 hours only)
  - Body weight and temperature
  - EKG (first 24 hours only)
  - Urine Output (from previous 24 hours)
  - Net fluid balance (record until ICU discharge or Day 28 - from previous 24 hours)
- **Clinical Laboratory Tests (first 24 hours only)**
  - CBC with differential
  - BUN, Creatinine
  - Na, K, Cl, HCO_3, Ca, Mg, Ionized Ca,PO_4
- **Respiratory**
  - Status – Ventilator support (yes/no)
  - If on Vent: Mode, FiO_2, Settings- TV, PEEP, RR
  - If on Vent with an arterial line: Arterial Blood Gas
  - If not Vented: Supplemental oxygen requirements
- **Renal Replacement Therapy status**
  - RRT status
- **Other (Event Driven)**
  - All the medications and blood products administered to the patient.
  - Pressor Usage (record until ICU discharge or Day 28)
  - Diagnostic/Therapeutic Procedures
  - Adverse Events
  - Microbiology/culture data (record until hospital discharge or Day 60)

6.5.2. DAY 28 POST RANDOMIZATION

This visit may be done via telephone call or office visit. Please record the following information on day 28 (+7 days) following enrollment:

- Blood pressure, heart rate and temperature (if known)
- Presence of mechanical ventilation (since last visit)
- Presence of renal replacement therapy (since last visit)
- Adverse events (since last visit)
- Serum creatinine (if an office visit takes place)

6.5.3. DAY 60 POST RANDOMIZATION

This visit may be done via telephone call or office visit. Please record the following information on day 60 (+7 days) following randomization:

- Blood pressure, heart rate and temperature (if known)
- Presence of mechanical ventilation (since last visit)
- Presence of renal replacement therapy (since last visit)
- ICU discharge date
- Hospital discharge date
- Adverse events (since last visit)
- Serum creatinine (if an office visit takes place)
- SF36v2 Quality of Life Questionnaire

7. TREATMENT INTERRUPTIONS – DISCONTINUATION CRITERIA

7.1. TREATMENT INTERRUPTION CRITERIA

The SCD is to be used continuously along with CRRT and changed every 24 hours or at anytime the circuit exhibits significant clotting that impairs the functionality of the circuit. For patients randomized to either arm of the study, each participating site will follow their institution’s protocol or standard of care in regard to replacement of the CRRT hemofilter and/or tubing.

In the event that a procedure is needed (e.g. CT-Scan, MRI), the CRRT or CRRT+SCD treatments may be interrupted for a maximum of 6 hours (cumulative) per 24 hour period. Cumulative interruption of greater than 6 hours in either the treatment or control arm will trigger the end of Observation Period and immediate initiation of the Follow-up period. These patients will not be removed from the study unless consent is withdrawn.

7.2. TREATMENT DISCONTINUATION CRITERIA

7.2.1. CLINICAL CRITERIA

Patients may be withdrawn from therapy (control or treatment) prior to hour 168 for a variety of reasons. When therapy is discontinued prior to hour 168, the follow-up period will immediately begin and data will continue to be collected per protocol.

The Principal Investigator will assign a primary reason for therapy termination utilizing the following categories, and if appropriate, one or more subcategories:

1. **Improvement in Clinical Status**
   - If a patient’s condition improves (e.g. renal condition has improved to the extent that CRRT can be discontinued or hemodynamic status has improved so that the dialysis modality

can be changed from CRRT to IHD) treatment may be discontinued before the 168 hour treatment period. The decision to discontinue treatment based upon improvement in clinical status will be made by the Principal Investigator.

2. **CRRT related medical events**
    - Such as persistent leukopenia, neutropenia or thrombocytopenia based upon PI's clinical assessment of relationship to control arm.

3. **SCD related medical events**
    - Such as persistent leukopenia, neutropenia or thrombocytopenia based upon PI's clinical assessment of relationship to SCD.

4. **CRRT Failure/Malfunction**
    - Failure of the CRRT device and/or circuit, including inability to maintain vascular access that requires treatment to be discontinued prior to hour 168 of therapy.

5. **SCD Failure/Malfunction**
    - Any failure of the SCD-ARF that requires treatment to be discontinued prior to hour 168 of therapy—e.g. clotting or evidence of leakage in the SCD-ARF or inability to maintain a patent circuit.

6. **Death, including withdrawal of life support**

7. **Concomitant Medical Conditions**
    - Need for medical, surgical or diagnostic procedures that necessitate discontinuation of CRRT or CRRT + SCD treatment for longer than the allowable 6 hours per 24 hour period.
8. **Other**
    - Any other reason that the Principal Investigator deems appropriate for discontinuation from the Study Observation period must be documented.

7.2.2. NON-CLINICAL

- **Withdrawal of participation from study observation period**

  Patients may withdraw their consent to participate in the study observation period (CRRT or CRRT + SCD) at any time. If the patient wishes to remain in the clinical study, the follow-up period will immediately begin at the termination of study observation period. All data will continue to be collected per protocol.

The Principal Investigator will notify the Sponsor's Chief Medical Officer or designee within 24 hours of withdrawal of consent.

- **Withdrawal of consent**
  If a patient or legal representative withdraws consent, all protocol related activities will be immediately discontinued. If study therapy (CRRT+SCD) is being administered at the time of withdrawal, normal procedures will be followed for CRRT+SCD therapy discontinuation (please see SCD Operator's Manual). No further study activity will be conducted.

  The Principal Investigator will notify the Sponsor's Chief Medical Officer or designee within 24 hours of withdrawal of consent.

8. CLINICAL TRIAL TERMINATION CRITERIA

The Sponsor reserves the right to terminate the clinical trial for safety or administrative reasons at any time. If the Sponsor, Principal Investigator, Data Safety Monitoring Board (DSMB), Institutional Review Board (IRB), or Food and Drug Administration (FDA) officials discover conditions during the study indicating that the trial or participation by a clinical site should be discontinued, this action may be taken after appropriate consultation between the Sponsor and the Investigators.

Conditions that may warrant discontinuation of the trial at a specific clinical site may include the following:

- Failure of the Investigator to enroll patients into the trial at an acceptable rate (as defined and agreed upon between the Investigator and the Sponsor).
- Failure of the Investigator to comply with the pertinent FDA regulations.
- Submission of false information by the Investigator from the research facility to the Sponsor or the FDA.

Conditions that may warrant discontinuation of the trial may include, but are not limited to, the following:

- Discovery of an unexpected, serious or unacceptable risk to the patients enrolled in the study.
- Decision on the part of the Sponsor to suspend or discontinue testing, evaluation or development of the study product at any time.

9. SAFETY

9.1. ADVERSE EVENTS

An Adverse Event is any sign, symptom, illness, clinically significant abnormal laboratory value or other adverse medical event that appears for the first time or worsens in a subject (control or treatment arm) during this clinical study, regardless of whether or not it is considered related to the device.

9.2. SERIOUS ADVERSE EVENTS

Any Adverse Event, whether considered study-treatment related or not, which fits any of the criteria below, is considered a serious adverse event (SAE):

- Results in death
- Is life-threatening (meaning that the patient was at risk of death at the time of the event; this does not refer to an event which might have caused death if it had occurred in a more severe form)
- Requires in-patient hospitalization or prolongs the existing hospitalization
- Is a persistent disability/incapacity
- Is a congenital anomaly or birth defect
- Is considered an important medical event by the Principal Investigator (e.g., surgery, return to ICU, emergency procedures)

9.3. UNANTICIPATED ADVERSE DEVICE EFFECT

Unanticipated adverse device effect (UADE) is any serious adverse effect on health or safety, any life-threatening problem or death caused by, or associated with a device, if that effect, problem, or death was not previously identified in nature, severity, or degree of incidence in the application; or any other unanticipated serious problem associated with a device that relates to the rights, safety, or welfare of subjects.

9.4. ANTICIPATED ADVERSE EVENT

Co-morbidities and symptoms/laboratory/physiological deviations normally associated with pre-existing conditions (e.g., diabetes, ASHD, other C-V conditions, pneumonia, dialysis shunt problems, neurologic deficits) are considered “anticipated adverse reactions.” The investigator is required to take special care in differentiating concomitant illness events from those related to the randomized therapy by use of patient history, relationship to treatment time and cartridge integrity, and other characteristics of clinical circumstances present at the time of the adverse experience, including drug interactions of concomitant medications and effects of surgical and/or medical procedures.

Adverse events associated with CRRT or underlying critical illness are also to be considered “anticipated”. Such events include, but are not limited to: thrombocytopenia, hyponatremia, hypokalemia, hypo- or hypercalcemia, hypo- or hyperglycemia, air embolism, hypotension, hemolysis, increased oxygenation requirements, leukopenia, arrhythmias, hypothermia, lactic acidosis, temporary decrease in cardiac output or cardiac index, disruption of skin integrity, bleeding, shock, bacteremia, hypotension, and death.

There are many anticipated events in the ICU patient that are not listed here. Any questions as to the expectedness of an adverse event will be discussed with CytoPherx’s CMO and/or the DSMB.

9.5. CAUSALITY

All randomized patients must have all AEs assessed for causality (probability that the AE may have been caused by the study treatment) by the Principal Investigator. The following definitions for causality assessment will be used in this study:

9.5.1. DEFINITELY RELATED

A clinical event, including a significant change in a laboratory test, that occurs in a plausible time relationship to the SCD treatment or other protocol-required activity, and which cannot be explained by concurrent disease or other drugs, chemicals, or procedures and that follows a clinically reasonable response upon withdrawal of the SCD treatment.

9.5.2. PROBABLY RELATED

A clinical event, including a significant change in a laboratory test, that occurs within a reasonable time sequence in relationship to the SCD treatment or other protocol-required activity that is unlikely to be attributed to concurrent disease, other drugs, chemicals, or procedures, and that follows a clinically reasonable response upon withdrawal of the SCD treatment.

9.5.3. POSSIBLY RELATED

A clinical event, including a significant change in a laboratory test, that occurs within a reasonable time sequence in relationship to the SCD treatment or other protocol-related activity that could also be explained by concurrent disease, drugs, chemicals, or procedures. The clinical course after withdrawal of the SCD treatment may be unclear with respect to the contribution of the SCD treatment to the AE.

9.5.4. UNRELATED

An AE, including a significant change in a laboratory test, that occurs with a temporal relationship to the SCD treatment or protocol-required activity that makes an association with the SCD treatment or study activity improbable, and in which other drugs, procedures or underlying disease(s) provide likely explanation.

9.6. SEVERITY – INTENSITY

The intensity of all adverse events should be evaluated using the following definitions:

9.6.1. MILD

An event that requires minimal clinical treatment or an adverse event requiring monitoring but no intervention or treatment; causes slight discomfort.

9.6.2. MODERATE

An event that requires non-routine intervention, (i.e., a new clinical treatment or diagnostic procedure), administered within an hour of the event; causes annoying discomfort.

9.6.3. SEVERE

An event requiring immediate intervention; causes significant discomfort.

9.7. ADVERSE EVENT REPORTING

Adverse event reporting requirements will be based on the time period in which the adverse event occurs.

9.7.1. SCREENING PERIOD (CONSENT THROUGH RANDOMIZATION)

Only adverse events, serious and not serious, directly related to the screening procedures will be capture during this period. Unrelated clinical adverse events that occur prior to randomization will be considered part of the medical history.

9.7.2. RANDOMIZATION THROUGH END OF STUDY

All adverse events that occur from the time of randomization/enrollment will be recorded as follows:

- Serious Adverse Events (identified in 9.2) will be recorded until Day 60 after randomization, death, or patient withdrawal of study consent, whichever occurs first.
- Non-serious adverse events will be recorded until Day 60 after randomization, death, patient withdrawal of study consent or until hospital discharge, whichever occurs first.

Any serious adverse event (regardless of relationship to the treatment) occurring from the time of enrollment/randomization through end of study must be reported to CytoPherx Inc, or designee, within 24 hours of knowledge of the event. This includes patients in the control arm.

Each clinical trial site will be supplied with written SAE reporting instructions, SAE reporting forms and contact information for reporting of serious adverse events.

For any AE that is ongoing at the time of the initial report, periodic follow-up information will be required until the adverse event is resolved or the patient is no longer in the study, whichever occurs first.

The Investigator is responsible for all adverse events reporting to the Institutional Review Board (IRB) according to the requirements of the IRB and for providing clinical trial monitors with all medical records needed to source-verify the adverse events.

10. CLINICAL EVENTS COMMITTEE

A clinical events committee (CEC) will be in place to review and adjudicate certain events that occur. At the onset of the trial, the CEC will establish a charter that outlines the criteria for which an event requires review, the method in which the reviews will take place, and the minimum documentation required for reviews. The CEC will be made up of physicians based on their clinical expertise and experience on CECs. They will have no other association with this trial or the sponsor. The CRO will have the primary role in composing and managing the CEC.

11. DATA SAFETY MONITORING BOARD

An independent Data Safety Monitoring Board (DSMB) will review safety results over the course of the study on a schedule set by CytoPherx, Inc. and the DSMB. In addition, the DSMB will be provided with one formal interim statistical analysis on the primary endpoint of 60 day mortality, as detailed in the statistical analysis section below. The DSMB will comply with the FDA Guidance: "Guidance for Clinical Trial Sponsors on the Establishment and Operation of Clinical Trial Data Monitoring Committees". The DSMB will be formed to provide scientific and medical feedback for the study.

The DSMB will focus on the following areas:

- *Performance* – to assess study conduct and compliance;
- *Effectiveness* – to assess mortality;
- *Safety* – to assess the incidence, severity, relationship, and timing of adverse events and to identify safety concerns; and
- *Context* – to assess the study relative to the AKI literature.

The DSMB will consist of a minimum of 5 independent experts including a minimum of 4 clinicians and a biostatistician. The DSMB will operate under a charter and will identify prospective data display specifications to conduct independent data reviews. The DSMB will make recommendations to the Sponsor to continue the study as planned or to stop the study due to safety or futility issues. The DSMB will meet to perform safety and effectiveness evaluations and to provide feedback to the Sponsor at least annually as well as after 25% and 50% of the cohort has completed 60 day follow-up. The DSMB reserves the right to request more frequent evaluations.

12. STATISTICAL ANALYSIS

12.1. GENERAL CONSIDERATIONS

The primary endpoint in this trial is time to all-cause mortality through 60 days post-randomization. The primary analysis is the assessment of difference between the experimental treatment (CRRT+SCD) and the control treatment (CRRT alone) on the primary endpoint, and the assessment of this difference will be carried out at an overall two-sided 0.05 level of significance. Statistical treatment comparisons on secondary endpoints will also be carried out at an overall two-sided 0.05 level of significance, unless otherwise specified. Statistical analyses will be carried out using SAS version 9.1 or higher, and other appropriate

statistical software if necessary.

12.2. DETERMINATION OF SAMPLE SIZE

The efficacy null and alternative hypotheses to be assessed are as follows:

$$H_0: \lambda_{C+S} = \lambda_C$$
vs.
$$H_1: \lambda_{C+S} \neq \lambda_C$$

where λ_{C+S} and λ_C are the true 60 day mortality hazard rates for the CRRT+SCD and CRRT alone groups, respectively. The null hypothesis will be tested at the overall two-sided 0.05 level of significance at 80% power. Under the assumptions:

1. $\lambda_C = 0.01155$ when time to mortality is measured in days (leading to a 50.0% mortality rate at 60 days for CRRT alone); $\lambda_{C+S} = 0.00718$ when time to mortality is measured in days (leading to a 35.0% mortality rate at 60 days for CRRT + SCD);

2. As described in the SCD-003 Protocol, CytoPherx has assumed a mortality rate of 50% for control and 35% for SCD.

   For the control mortality estimate, a 2004 publication [20] was used which summarized mortality data for subjects with acute renal failure (ARF) requiring renal replacement therapy (RRT) in the intensive care unit. A table from this publication is reproduced below (Table 9). In this table, the mortality of subjects who received CRRT ("Dialysis") is compared to those who did not ("% Mortality No Dialysis"). A weighted average was calculated by weighting the observed value for "% Mortality Dialysis" by the number of subjects enrolled. We found that the weighted mortality of subjects who received CRRT was 58.3%, based on this literature analysis of 5,810 subjects.

Variable	Ref.	Year	*N*	Location	% Mortality No Dialysis	% Mortality Dialysis
Brivet et a l	[26]	1996	360	Multicenter, France	43%	64%
Liano et al	[5]	1998	748	Multicenter, Spain	53%	79%
De Mendonca et al	[27]	2000	1411	16 countries, Europe	NA	44%
Silvester et al	[28]	2001	299	Multicenter, Australia	NA	47%
Metniz et al	[7]	2002	839	Multicenter, Austria	39%	63%
Clermont et al	[29]	2002	254	Pittsburgh, USA	23%	57%
Guerin et al	[30]	2002	587	Multicenter, France	NA	71%
Metcalfe et al	[31]	2002	89	Multicenter, Scotland	NA	74%
Mehta et al	[32]	2002	605	Multicenter, California USA	39%	61%
PICARD	[20]	2003	618	Multicenter, USA	24%	45%

Table 9 Several recent studies of ARF in the ICU

In our proposed pivotal study, the assumed 60-day mortality rate for subjects receiving CRRT only (control subjects) is 50%. This is a conservative estimate

since, based on the literature, the actual control mortality rate might be expected to be slightly higher.

The observed 60 day mortality rate for SCD therapy was 29.2% in the Pilot study with data available for 24 subjects. For the pivotal study, we have increased the assumed mortality rate to 35% to allow for variability in response across a broader population of subjects and treatment centers;

3. The statistical test to compare treatments is the log-rank test;

4. The study will be carried out using an adaptive design approach; specifically, there will be one formal interim analysis on mortality using the log-rank test after 50% of the subjects achieve 60 day follow-up (or would have achieved 60 day follow-up had they not died or prematurely withdrawn from the study); treatments will be compared via the log-rank test at this interim analysis using the O'Brien-Fleming two-sided significance levels (0.00305) for the interim analysis (leaving a two-sided significance level of 0.049 for the final analysis); only subjects achieving 60 day follow-up (or who would have achieved 60-day follow-up had they not died or prematurely withdrawn) will be used in the interim analysis; note that if significance is shown in the interim analysis, a decision to stop the trial will only be made after consultation with the FDA; if significance is not shown at this interim analysis, then the DSMB will be provided with sample size re-calculations required to maintain at least 80% power for a statistically significant CRRT+SCD effect by the end of the study, conditioned on the interim results (details are provided below).

Then the required evaluable sample size is 166 subjects per group. To account for attrition, which is expected to be minimal, 172 subjects per group will be randomized. The sample size was calculated using the PASS 2008 software, taking into account the fact the study may be stopped for overwhelming efficacy at the interim analysis using the log-rank test at the O'Brien-Fleming significance level of 0.00305.

12.3. ANALYSIS POPULATIONS

Intent-to-Treat (ITT) Analysis Set: All randomized subjects. **This is the primary analysis set on which primary and secondary efficacy endpoints will be evaluated.** All subjects will be analyzed according to the treatment to which they were randomized.

Per-Protocol Analysis Set: The subset of subjects from the ITT analysis set in whom randomized treatment was initiated, who received the treatment to which they were randomized, who did not violate the inclusion/exclusion criteria, and who did not prematurely withdraw from the study (except due to death) by 60 days post-randomization.

Safety Analysis Set: The subset of ITT subjects in whom treatment was initiated.

This is the primary analysis set for the safety analysis. Subjects will be analyzed according to treatment received.

12.4. DEMOGRAPHICS AND BASELINE CHARACTERISTICS

Baseline characteristics will be descriptively summarized for all subjects by treatment group for the ITT analysis set. Continuous measures will be summarized with sample size, mean, median, standard deviation, minimum and maximum; categorical measures will be presented with the counts and percents of subjects in each category. No statistical tests comparing treatments at baseline will be performed.

12.5. PRIMARY ENDPOINT

The primary endpoint in this trial is time to mortality through 60 days post-randomization. The Kaplan-Meier curve of time (days) to mortality through 60 days post-randomization will be presented, as will the Kaplan-Meier estimate of the 60 day mortality rate. In addition, the raw number and percentage of subjects who died by 60 days will be presented by treatment group. Treatment groups will be compared on time to mortality through 60 days using the log-rank test. The analysis will be carried out for the ITT (primary) and Per-Protocol (secondary) analysis set. It is anticipated there will be few randomized subjects who prematurely withdraw from the trial. Subjects who do not die will be censored at 60 days or at the last known time alive, whichever is earlier.

Due to one formal interim efficacy analysis following which the DSMB may recommend an increase in sample size (based on the observed treatment difference seen at the interim analysis) beyond the initially planned 172 randomized patients (see Section 12.8 for details), the final analysis comparing treatments on the primary endpoint does not use the conventional log-rank test p-value to determine if a statistically significant treatment difference exists. Instead it uses a weighted test statistic, T^*, proposed by Cui, Hung and Wang [33] in which the independent test statistics of the two stages (interim and final) are combined by pre-specified weights, w_1 and $w_2 = 1 - w_1$, where w_1 is the proportion of the total *initially* planned number of patients at which the interim analysis is performed. In the present case the trial is designed for 172 randomized subjects per group with an interim analysis after 50% of the patients achieve 60 day follow-up (or would have achieved 60 day follow-up had they not died or prematurely withdrawn). Thus, the planned information time proportions are $w_1 =$ 50% for first stage and $w_2 = 1 - w_1 =$ 50% for the second stage.

Hence, two log-rank tests will be performed. The first (interim) log-rank test will be based on all patients who achieved 60-day follow-up (or would have achieved 60 day follow-up had they not died or prematurely withdrawn) at the time of the interim analysis. The log-rank test Z statistic for treatment effect from this model is denoted as $Z^{(1)}$. The second log-rank test will be based on all patients in the full analysis set at the end of the study. The log-rank Z statistic for treatment effect from this model is denoted as Z^*. Then define

$$Z^{(2)} = \frac{1}{\sqrt{1-\frac{t_1}{t^*}}} Z^* - \frac{\sqrt{\frac{t_1}{t^*}}}{\sqrt{1-\frac{t_1}{t^*}}} Z^{(1)}$$

where t_1 = ratio of observed number of patients at the interim analysis to the *initially planned* total required number of randomized patients (172); and t^* = ratio of the number of randomzied patients at the end of the trial to the initially planned total number of randomized patients (172). $Z^{(2)}$ is independent of $Z^{(1)}$ and captures the information about the treatment effect in Z^* that is not in $Z^{(1)}$ [34].

At the final analysis, the test statistic used to compare treatments will be the weighted sum of $Z^{(1)}$ and $Z^{(2)}$ as defined above, using the square root of the planned proportions (square roots of w_1 and w_2 where $w_1 = w_2 = 1- w_1 = 0.5$) as the weights, respectively. Specifically, the weighted test statistic for the final analysis, T^*, is defined as:

$$T^*=\text{sqrt}(w_1)Z^{(1)}+\text{sqrt}(w_2)Z^{(2)}$$

This combined test statistic T^* will be used to test the treatment effect with respect to the primary outcome at the final analysis. This test statistic controls the overall type I error in the presence of a sample size increase. This has been confirmed by computer simulations.

The above is the primary analysis on the primary endpoint. Additional analysis on the primary endpoint will be as follows: to account for premature withdrawal from the study, the following analysis will also be performed on the ITT analysis set as a secondary sensitivity analysis: For patients who prematurely withdrew prior to 60 days post-randomization, missing mortality status will be imputed before carrying out the treatment comparisons. Imputation of missing mortality status will be carried out using the multiple-imputation logistic regression procedure using clinically relevant baseline characteristics as risk factors in the regression model (these characteristics will be fully specified in the study's statistical analysis plan). A total of 10 imputed data sets will be generated, and logistic regression comparing treatments on 60 day mortality status will be run on each data set. An assessment of overall treatment difference on 60 day mortality will then be generated from combining the logistic regression estimate of the treatment effect and the estimate of its standard error across the 10 data sets. This will not be done at the interim analysis, but only at the final analysis, at the same final significance level as will be used for the primary analysis.

For the primary (non-imputed) analysis on the ITT data set, the raw Kaplan-Meier estimates of mortality rate will be presented for each treatment group within each study center. An assessment of treatment-by-study center interaction effect will be carried out using Cox proportional hazards regression containing the main effects of treatment and center and the interaction effect of treatment-by-center. A treatment-by-center interaction effect that is not significant at the 0.15 level of

significance, or that is significant but only quantitative in nature, will not preclude pooling of patients across study centers for the primary endpoint analysis. Prior to breaking the randomization blind, study centers with less than 10 subjects will be pooled with other study centers located in similar geographic regions.

All enrolled patients will receive standard intensive care treatment for patients undergoing CRRT or CRRT+SCD. Any mode of CRRT will be allowed (CVVH, CVVHD, and CVVHDF) and the modality of CRRT is not expected to affect performance of the SCD. The modality-by-treatment interaction will be assessed. Specifically, for the primary (non-imputed) analysis on the ITT data set, the raw Kaplan-Meier estimates of mortality rate will be presented for each treatment group within each modality. An assessment of treatment-by-modality interaction effect will be carried out using Cox proportional hazards regression containing the main effects of treatment and modality and the interaction effect of treatment-by-modality. A treatment-by-modality interaction effect that is not significant at the 0.15 level of significance, or that is significant but only quantitative in nature, will not preclude pooling of patients across modalities for the primary endpoint analysis.

12.6. SECONDARY ENDPOINTS

- Renal Replacement Therapy dependency at day 60
- Time-to-Mortality through day 28
- Number of ventilator free days (VFD) at day 28
- Time-to-Mortality through day 60 for the Severe Septic patient(s)

Definitions for secondary endpoints are provided in Appendix D

For renal replacement therapy dependency, treatments will be compared on the number and percentage of patients with the incidence of the event using the Z test for proportions or the continuity-corrected z-test for proportions, depending on event rate. For time-to-mortality through day 28 and for time to mortality through day 60 for the severely septic patient sub-population, treatments will be compared using the log-rank test. For number of VFD at day 28, the sample size, mean, standard deviation, median, quartiles, and minimum and maximum will be presented for each treatment group. Treatment groups will be compared using the two-sample t-test. Health Economic endpoints are still under development will be discussed in a separate plan. These endpoints are not intended to support a PMA application.

In order to control the overall two-sided level of significance at 0.05 across the secondary endpoints, the Benjamini-Hochberg procedure will be used. For time-to-mortality analysis, patients who did not die will be censored at the time of analysis (day 28 or day 60) or at the time of premature withdrawal, whichever is earlier. Otherwise, given the secondary nature of these endpoints, no imputation for missing data will be performed; i.e., patients prematurely withdrawing prior to the time point at which the endpoint is measured will be excluded from the

analysis on that endpoint.

12.7. ADVERSE EVENT ANALYSIS

Adverse event analysis will be performed on the safety population. Adverse Events (AEs) will be mapped to preferred term and body system using the Medical Dictionary for Regulatory Activities (MedDRA). Adverse Event listings will be provided for all AEs, SAEs, and all UADEs leading to study discontinuation.

The number and percentage of patients experiencing AEs will be summarized by treatment group overall and for each System Organ Class and Preferred term. This analysis will be repeated for Serious AEs and for any AEs leading to treatment/study discontinuation. AEs will also be tabulated by maximum severity experienced. Treatments will be compared on incidence of AEs using Fisher's Exact Test.

12.8. INTERIM ANALYSIS/ADAPTIVE DESIGN

There will be one formal interim efficacy analysis on the primary endpoint of time to all-cause mortality by 60 days, to be inspected by the DSMB. This analysis will be performed on the ITT analysis set after 50% of the planned number of patients achieved 60 day follow-up (or would have achieved 60 day follow-up at the time if the patient did not die or prematurely withdraw). Using the Lan-DeMets function with the O'Brien-Fleming philosophy, the two-sided treatment comparison significance levels to be used at the interim and final analysis are 0.00305 and 0.049, respectively. If the DSMB recommends stopping the study at the interim analysis (i.e., if the two- sided p-value for the primary endpoint is below 0.00305 at the interim analysis), a decision to stop the study will only be made after consultation with the FDA.

If the DSMB does not recommend stopping the study at the interim analysis for overwhelming efficacy, the DSMB may recommend stopping the study for futility if the interim estimate of survival for the CRRT alone group is larger than that for the CRRT + SCD group (in which case, no further enrollment of patients will be carried out, but patients already enrolled at the time of the decision to stop for futility will continue to be followed for 60 days. Otherwise, the DSMB will be presented with a proposed increase in sample size for achieving a statistically significant treatment difference on the primary endpoint by the time of the final analysis. The sample size re-estimation will be performed based on the approach suggested by Cui, Hung and Wang (1999) using the ratio of the initial estimate of effect size, E_0, which is the difference between Kaplan-Meier estimates of 60 day mortality rate (CRRT + SCD minus CRRT alone) for the initial sample size determination used for study design, and the observed effect size, E, which is the treatment difference in estimated Kaplan-Meier estimate of the 60 day mortality rate at the time of the interim analysis.

The number of patients initially estimated, N_o (=172), is readjusted to determine the new total number of required patients, N, using the following formula:

$$N = \left|\frac{E_o}{E}\right|^2 N_o$$

This method will provide the required number of patients for the second stage of the study. In case the sample size re-assessment results in an increase of the number of subjects, the DSMB may recommend changing the study accordingly (if however the increase in sample size is more than twice the number of originally planned subjects, the DSMB may recommend continuing the study until twice the number of originally planned patients are randomized). In case the sample size re-assessment results in an adaption to less than the original planned sample size, the study will be continued as originally planned (until 172 subjects are randomized).

The statistical methodology used to compare treatments at the interim and final analysis is discussed above. The overall type I error of 5% is controlled as has been confirmed by simulations.

The DSMB will periodically review safety data. A DSMB charter will be written outlining the safety data to be inspected periodically by the DSMB. The DSMB may recommend stopping the study for safety issues, and this recommendation does not need to necessarily be based on statistical significance.

12.9. EXPLORATORY ANALYSIS

In addition a number of exploratory analyses will be conducted. No formal statistical hypothesis tests will be conducted. Predictors of survival will be assessed using Cox proportional hazards regression utilizing univariate and multivariate models to assess association of selected parameters with the primary outcome. Some parameters to be assessed may include gender, age, race/ethnicity, presence of co-morbidities, type of CRRT modality, SOFA score and RIFLE predicted outcomes. Additional regression models may include interaction terms for the selected parameters to assess for evidence of a differential treatment effect. Other exploratory analyses on additional outcome measures will describe the following outcomes in the two randomized groups: infection as defined in Appendix D, pressor free days,recovery of renal function, SCD integrity, ICU LOS, Hospital LOS, duration of Renal Support, CRRT circuit life analysis and fluid removal analysis. Continuous outcomes will be summarized with sample size, mean, median, standard deviation, minimum, maximum and 95% confidence intervals; categorical outcomes will be presented with the counts and percents of subjects in each category along with 95% confidence limits. Additional, non pre-specified exploratory analyses may also be conducted.

13. ECONOMIC AND QUALITY OF LIFE (QOL) EVALUATION

13.1. QOL AND ECONOMIC STUDY OBJECTIVES

The specific objectives for the QOL and economic study are as follows:

- To assess health-related quality of life and health state utility between study groups 60 days after randomization.
- To estimate the costs of the hospital admissions in which renal replacement therapy is initiated and compare between groups.
- To measure major health care resource utilization from randomization to 60 days and compare between groups.
- To estimate total health care costs at 60 days and compare between groups.
- To estimate long-term cost effectiveness of renal replacement therapy for acute kidney injury using the SCD, relative to providing renal therapy without the SCD.

14. DATA COLLECTION – STUDY MONITORING AND AUDIT

The Principal Investigator is responsible for assuring the accuracy and completeness of all study documentation. Monitoring will be conducted within ICH/GCP Guidelines and CytoPherx, Inc. Clinical Monitoring Plan to ensure the following:

- The facilities continue to be acceptable.
- The protocol is appropriately followed.
- Any agreed upon changes to the protocol have been approved by the IRB and approval has been received in writing by CytoPherx, Inc.
- Accurate, complete and current records are maintained and available for all patients.
- For each subject. collection of protocol-required data and entry of that data into the CRFs must be completed as soon as reasonably possible and shall not be more than 90 days following that subject's last day in the study without permission from CytoPherx, Inc.
- The information recorded and submitted to CytoPherx, Inc. is representative of the patient's record and other supporting documentation.
- Accurate, complete and timely adverse event reports for serious adverse events are submitted to CytoPherx, Inc.
- The Principal Investigator continues to assume primary responsibility for the study.

The Investigator or designee must, upon request, provide to the Clinical Research Associate (CRA), Quality Assurance Auditor, or FDA Investigator the necessary study records for a thorough review of the study's progress. These records include, but are not limited to, original documents and records, such as hospital and clinic charts, consent forms, and laboratory records. The Principal Investigator is required to notify the Sponsor immediately of the following:

- Withdrawal of IRB Approval
- Any deviations from the protocol
- Notification that Informed Consent was not obtained or consent was withdrawn
- Any other instance in which the Investigator or Sponsor deems it necessary

15. INVESTIGATOR'S STATEMENT OF RESPONSIBILITY

By my signature, I confirm that my staff and I have carefully read and understand this protocol and agree to comply with the conduct of the specified therein, In particular we have agreed to:

- Conduct the study according to the protocol, amendments, and study guides.
- Obtain Institutional Review Board approval of the study, any amendments to the study and periodic-re-approval, as required.
- Obtain written consent from each study participant or their legal representative.
- Report all serious adverse events to CytoPherx, Inc and to the IRB, as required by the protocol and IRB regulations.
- Assure access by study monitors to original source documents.
- Cooperate fully with any study-related GCP audit as performed by CytoPherx, Inc, their designee, or the Food and Drug Administration (FDA).
- Maintain confidentiality and assure security of confidential documents such as the protocol, consent form, case report form, SCD Operator's Manual, final study reports, manuscript, and/or unpublished data and correspondence.

____________________________________ _______________

Principal Investigator Signature Date

____

Printed Name

16. REFERENCES

1. Kjellstrand CM, Gornick C, Davin T. Recovery from acute renal failure. Clin Exp Dialys Apher 1981;5(1&2):143–161.

2. Hall JW, Johnson WJ, Maher FT, Hunt JA, Immediate and Long-Term Prognosis in Acute Renal Failure. Ann Intern Med 1970;73: 515–521.

3. Kjellstrand CM, Berkseth RO, Klinkmann H. Treatment of acute renal failure. In: Diseases of the Kidney. Eds. Schrier RW and Gottschalk CW. Boston: Little Brown, 1988; 1501–1534.

4. Van Den Noortgate D, Mouton V, Lamot C, Van Nooten G, Chondt A, Vanholder R, Afschrift A, Lameire N. Outcome in a post-cardiac surgery population with acute renal failure requiring dialysis: Does age make a difference? Nephrol Dial Transplant 2003; 18:732–736.

5. Liaño F, Junco E, Pascual J, Madero R, Verde E. The spectrum of acute renal failure in the intensive care unit compared with that seen in other settings. Kidney Int Suppl 1998;53 Supplement 66:S16-S24.

6. Uchino S, Kellum JA, Bellomo R, et al. Acute renal failure in critically ill patients: a multinational, multicenter study. JAMA 2005;294: 813-818.

7. Metnitz PG, Krenn CG, Steltzer H, et al. Effect of acute renal failure requiring renal replacement therapy on outcome in critically ill patients. Crit Care Med 2002; 30:2051-2058.

8. McCarthy JT. Prognosis of patients with acute renal failure in the intensive care unit: a tale of two eras. Mayo Clin Proc 1996;71: 117-126.

9. Star RA. Treatment of acute renal failure. Kidney Int 1998;54: 1817-1831.

10. Simmons EM, Himmelfarb J, Sezer MT, Chertow GM, Mehta RL, Paganini EP, Soroko S, Freedman S, Becker K, Spratt D, Shyr Y, Ikizler TA, et al. Plasma cytokine levels predict mortality in patients with acute renal failure. Kidney Int 2004; 65:1357–1365.

11. Mutunga M, Fulton B, Bullock R, Batchelor A, Gascoigne A, Gillespie JI, Baudouin SV. Circulating endothelial cells in patients with septic shock. Am J Respir Crit Care Med 2001; 163:195–200.

12. Himmelfarb J, McMonagle E, Freedman S, Klenzak J, McMenamin E, Le P, Pupim LB, Ikizler TA, et al. Oxidative stress is increased in critically ill patients with acute renal failure. J Am Soc Nephrol 2004; 15:2449–2456.

13. Kaneider NC, Leger AJ, Kuliopulos A. Therapeutic targeting of molecules involved in leukocyte-endothelial cell interactions. FEBS J 2006;273: 4416–4424.

14. Maroszynska I, Fiedor P. Leukocytes and endothelium interaction as rate limiting step in the inflammatory response and a key factor in the ischemia-reperfusion injury. Ann Transplant 2000;5(4):5–11.

15. Ware, LB, Matthay, MA. The acute respiratory distress syndrome. N Engl J Med, 342(18), 1334-49, 2000

16. Mehta RL. Anticoagulation Strategies for Continuous Renal Replacement Therapies: What Works? Amer Jour Kidney Dis 1996;28(5):S8-S14.

17. Holt AW, Bierer P, Bersten AD, Bury LK, Vedig AE. Continuous renal replacement therapy in critically ill patients: monitoring circuit function. Anaesth Intensive Care 1996;Aug:24(4):423-429.

18. Cubattoli L, Teruzzi M, Cormio M, Lampati L, Pesenti A. Citrate anticoagulation during CVVH in high risk bleeding patients. Int J Artif Organs 2007;30(3):244–252.

19. Nurmohamed SA, Vervloet MG, Girbes ARJ, Ter Wee PM, Groeneveld ABJ. Continuous venovenous hemofiltration with or without predilution regional citrate anticoagulation: A prospective study. Blood Purif 2007;25:316–323.

20. Mehta RL, Pascual MT, Soroko S, et al. Spectrum of acute renal failure in the intensive care unit: the PICARD experience: Kidney Int 2004;66:1613-21.

21. Ding F, Yevzlin A et al. (In Press). The Effects of a Novel Therapeutic Device on Acute Kidney Injury Outcomes in the Intensive Care Unit: A Pilot Study : ASAIO Journal.

22. Windeløv NA, Ostrowski SR, Perner A, Johansson PI: Transfusion requirements and clinical outcome in intensive care patients receiving continuous renal replacement therapy: comparison of prostacyclin vs. heparin prefilter administration. Blood Coagul Fibrinolysis. 2010 Jul;21(5):414-9

23. Rodriguez S, Chora A, Goumnerov B, Mumaw C, Goebel WS, Fernandez L, Baydoun H, HogenEsch H, Dombkowski DM, Karlewicz CA, Rice S, Rahme LG, Carlesso N: Dysfunctional expansion of hematopoietic stem cells and block of myeloid differentiation in lethal sepsis. Blood. 2009 Nov 5;114(19):4064-76. Epub 2009 Aug 20

24. Bihorac A, Ross EA Continuous Venovenous Hemofiltration with Citrate-Based Replacement Fluid: Efficacy, Safety, and Impact on Nutrition. Am J Kidney Dis 46:908-918, 2005

25. VA/NIH Acute Renal Failure Trial Network, Palevsky PM, Zhang JH, O'Connor TZ, Chertow GM, Crowley ST, Choudhury D, Finkel K, Kellum JA, Paganini E, Schein RM, Smith MW, Swanson KM, Thompson BT, Vijayan A, Watnick S, Star RA, Peduzzi P. Intensity of renal support in critically ill patients with acute kidney injury. N Engl J Med. 2008 Jul 3;359(1):7-20.

26. Brivet FG, Kleinknecht DJ, Loirat P, Landais PJ: Acute renal failure in intensive care units-causes, outcome, and prognostic factors of hospital mortality: A prospective, multicenter study. French Study Group on Acute Renal Failure. *Crit Care Med* 24:192-198, 1996

27. De Mendonca A, Vincent J, Suter P, *et al*: Acute renal failure in the ICU: Risk factors and outcome evaluated by the SOFA score. *Intensive Care Med* 26:915-921, 2000
28. Silvester W, Bellomo R, Cole L: Epidemiology, management, and outcome of severe acute renal failure of critical illness in Australia. *Crit Care Med* 29:1910-1915, 2001

29. Clermont G, Acker CG, Angus DC, *et al*: Renal failure in the ICU: Comparison of the impact of acute renal failure and end-stage renal disease on ICU outcomes. *Kidney Int* 62:986-996, 2002

30. Guerin C, Girard R, Selli J, Ayzac L: Intermittent versus continuous renal replacement therapy for acute renal failure in intensive care units: Results from a multicenter prospective epidemiological survey. *Intensive Care Med* 28:1411-1418, 2002

31. Metcalfe W, Simpson M, Khan IH, *et al*: Acute renal failure requiring renal replacement therapy: Incidence and outcome. *QJM* 95:579-583, 2002
32. Mehta RL, Pascual MT, Gruta CG, *et al*: Refining predictive models in critically ill patients with acute renal failure. *J Am Soc Nephrol* 13:1350-1357, 2002

33. Cui, L., Hung, HM., and Wang, SJ "Modification of sample size in group sequential clinical trials." *Biometrics* 55: 853-857 (1999)

34. Lawrence, J. and Hung, HMJ. Estimation and Confidence Intervals after Adjusting the Maximum Information. *Biometrical Journal*, 45 (2): 143-152 (2003).

35. Vincent JI, Moreno R, Takala J, Willatts S, DeMendonca A, Bruining H, Reinhart CK, Suter PM, Thijs LG. The SOFA (Sepsis related Organ Failure Assessment) score to describe organ dysfunction/failure.Intensive Care Med 1996; 22:707-710.

36. Fein, Alan. <u>Sepsis and Multiorgan Failure</u>. Baltimore: Williams and Wilkins, 1997.

17. APPENDICES

APPENDIX A
SEQUENTIAL ORGAN FAILURE SCORING SYSTEM (SOFA) [35]

ORGAN SYSTEM	MEASURE
Respiration	PaO_2 to FiO_2 Ratio
Coagulation	Platelet Count
Liver	Serum Bilirubin
Cardiovascular	Hypotension
Central nervous system	Glasgow Coma Score
Renal	Serum Creatinine or Urine Output

MEASURE	FINDING	POINTS
PaO_2 to FiO_2 Ratio (please see Pulmonary System Conversion Table for Non-ventilated Pts)	≥ 400 mmHg	0
	300 – 399 mmHg	1
	200 – 299 mmHg	2
	100 – 199 mmHg	3
	< 100 mmHg	4
Platelet Count	≥150 x $10^3/mm^3$	0
	100 – 149 x $10^3/mm^3$	1
	50 – 99 x $10^3/mm^3$	2
	20 – 49 x $10^3/mm^3$	3
	< 20 x $10^3/mm^3$	4
Serum Bilirubin	< 1.2 mg/dL	0
	1.2 – 1.9 mg/dL	1
	2.0 – 5.9 mg/dL	2
	6.0 – 11.9 mg/dL	3
	≥12.0 mg/dL	4
Hypotension	Mean arterial pressure ≥70 mmHg	0
	Mean arterial pressure < 70 mmHg no pressor agents used	1
	Dobumatine any dose	2
	Dopamine ≤ 5 µg per kg per min	2
	Dopamine > 5 – 15 µg per kg per min	3
	Dopamine > 15 µg per kg per min	4
	Epinephrine ≤ 0.1 µg per kg per min	3
	Epinephrine > 0.1 µg per kg per min	4
	Norepinephrine ≤ 0.1 µg per kg per min	3
	Norepinephrine > 0.1 µg per kg per min	4

SEQUENTIAL ORGAN FAILURE SCORING SYSTEM (SOFA)
(Continued)

MEASURE	FINDING	POINTS
Glasgow Coma Score	15	0
	13 – 14	1
	10 – 12	2
	6 – 9	3
	3 – 5	4
Serum Creatinine or Urine Output	Serum Creatinine < 1.2 mg/dL	0
	Serum Creatinine 1.2 – 1.9 mg/dL	1
	Serum Creatinine 2.0 – 3.4 mg/dL	2
	Serum Creatinine 3.5 – 4.9 mg/dL	3
	Serum Creatinine > 5.0 mg/dL	4
	Urine Output 200 – 499 mL per day	3
	Urine Output < 200 mL per day	4

Conventions used for the above table include:

- PaO_2 is in mmHg and FiO_2 in percent from 0.21 to 1.00.
- Adrenergic agents as administered for at least 1 hour with doses in µg per kg per min.
- A score of 0 indicates normal and a score of 4 indicates most abnormal.
- Data can be collected and the score calculated daily during the course of the admission.
- Mean systemic arterial pressure = systolic arterial pressure + twice the diastolic arterial pressure divided by 3. All values should be in mmHg.
- Total Modified SOFA score = SUM (points for all 6 measures)

SEQUENTIAL ORGAN FAILURE SCORING SYSTEM (SOFA)
(Continued)

Interpretation:
1. Minimum total score: 0
2. Maximum total score: 24
3. The higher the organ scores the greater the organ dysfunction.
4. The higher the total scores the greater the multi-organ dysfunction.

	**MORTALITY RATE BY MODIFIED SOFA SCORE**				
**ORGAN SYSTEM**	**0**	**1**	**2**	**3**	**4**
Respiratory	20%	27%	32%	46%	64%
Cardiovascular	22%	32%	55%	55%	55%
Coagulation	35%	35%	35%	64%	64%
CNS	26%	35%	46%	56%	70%
Liver	32%	34%	50%	53%	56%
Renal	25%	40%	46%	56%	64%

GLASGOW COMA SCORE

The Glasgow Coma Score (GCS) is scored between 3 and 15, 3 being the worst and 15 the best. It is composed of the three parameters listed below:

Best Eye Response (4)
1. No eye opening
2. Eye opening to pain
3. Eye opening to verbal command
4. Eyes open spontaneously

Best Verbal Response (5)
1. No verbal response
2. Incomprehensible sounds
3. Inappropriate words
4. Confused
5. Oriented

Best Motor Response (6)
1. No motor response
2. Extension to pain
3. Flexion to pain
4. Withdrawal from pain
5. Localizing pain
6. Obeys commands

A Coma Score of 13 or higher correlates with a mild brain injury, 9 to 12 is a moderate injury and 8 or less a severe brain injury.

SEQUENTIAL ORGAN FAILURE SCORING SYSTEM (SOFA)
(Continued)

PULMONARY SYSTEM CONVERSION TABLE

**O_2 Saturation Conversion Table**		**Conversion Table for FiO_2**
Pulse oximetry O_2 saturation may be used for calculating PaO_2/FiO_2 ratio when ABG not available		When measured on mask or nasal cannula

SaO_2 (%)	Calculated PaO_2
80	44
81	45
82	46
83	47
84	49
85	50
86	52
87	53
88	55
89	57
90	60
91	62
92	65
93	69
94	73
95	79
96	86
97	96
98	112
99	145

Nasal Cannula	
100% O_2 Flow Rate (L/min)	FiO_2 (%)
1	24
2	28
3	32
4	36
5	40
6	44
Oxygen Mask	
100% O_2 Flow Rate (L/min)	FiO_2 (%)
5-6	40
6-7	50
7-8	60
9	90
10	99+
Mask with Reservoir Bag	
100% O_2 Flow Rate (L/min)	FiO_2 (%)
6	60
7	70
8	80

APPENDIX B

DEFINITIONS OF ACUTE KIDNEY INJURY/ACUTE TUBULAR NECROSIS

Acute Kidney Injury/Acute Tubular Necrosis

Acute Kidney Injury (AKI) is sudden loss of the ability of the kidneys to excrete wastes, concentrate urine, and conserve electrolytes.

Acute tubular necrosis (ATN) is defined in this protocol as AKI due to hemodynamic or toxic etiologies. These conditions are found in circumstances of acute ischemic or nephrotoxic injury and conform to the following criteria:

Oliguria: average < 20 mL/hour for > 6–12 hours ***OR***

Serum Creatinine: ≥ to an increase of 2 mg/dL for males over a period of ≤ 4 days or ≥ to an increase of 1.5 mg/dL in females over a period of ≤ to 4 days
(Note: pre-renal, hepatorenal, vascular, interstitial, glomerular, and obstructive etiologies are excluded on clinical or other diagnostic grounds.)

ATN develops predominantly due to the injury and necrosis of HPTC (Human Proximal Tubular Cells). ATN is caused by ischemia of the kidneys or by exposure to nephrotoxic agents. Risks for ATN include injury or trauma with resulting damage to the muscles, recent major surgery, blood transfusion reaction, septic shock or other forms of shock, and severe hypotension longer than 30 minutes.

Any condition that causes a reduction in the amount of blood being pumped by the heart may cause ATN. Liver disease and damage caused by diabetes mellitus (diabetic nephropathy) may predispose a person to the condition. ATN can also be caused by exposure to nephrotoxic agents (e.g., aminoglycoside antibiotics), antifungal agents (e.g., amphotericin), medications to prevent rejection of transplanted organs (e.g., cyclosporine), dye used for radiographic studies, and other toxins. In SCD studies, only patients with ATN due to hemodynamic or toxic etiologies are eligible for study participation. As noted above, pre-renal, hepatorenal, vascular, interstitial glomerular and obstructive etiologies are excluded on clinical or other diagnostic grounds.

APPENDIX C
DEFINITIONS OF SEPSIS AND SEVERE SEPSIS [36]

A. SEPSIS

Sepsis is defined in this protocol as a condition which has clinical evidence suggestive of infection, plus signs of a systemic response to infection with all of the following:

**Tachypnea**	**> 20 breaths/min[1]**
**Tachycardia**	**> 90 beats/min**
**Hyper/Hypothermia**	**> 38.4˚C or < 35.6˚C**

[1] If the patient is mechanically ventilated, > 10L/min.

B. SEVERE SEPSIS

Severe Sepsis is defined in this protocol as SEPSIS with one or more signs of organ dysfunction, such as:

**Metabolic acidosis**	**Higher than upper limit**
**Acute encephalopathy**	
**Oliguria**	**<0.5 ml/kg for at least one (1) hour**
**Hypoxemia or DIC[2] or hypotension**	**PaO_2/fiO_2 <280[3]**<br>**<90 mmHg (or a 40 mmHg decrease below baseline)**

[2] Disseminated intravascular coagulation
[3] If the patient is mechanically ventilated, >10L/min

APPENDIX D
DEFINITIONS

RENAL REPLACEMENT THERAPY (RRT) DEPENDANCY AT DAY 60

RRT dependency at day 60 is defined as patient not receiving any form of intermittent or continuous renal replacement therapy at 60 days post enrollment in the study with no plans for additional intermittent or continuous renal replacement therapy.

NUMBER OF VENTILATOR FREE DAYS (VFD) AT DAY 28

Ventilator Free Days (VFD) to day 28 is defined as the number of days of unassisted breathing to day 28 after randomization, assuming a patient survives for at least two consecutive calendar days after initiating unassisted breathing and remains free of assisted breathing. If a patient returns to assisted breathing and subsequently achieves unassisted breathing prior to day 28, VFD will be counted from the end of the last period of assisted breathing to day 28 unless a period of assisted breathing was less than 24 hours and the purpose of assisted breathing was a surgical procedure. If the patient is receiving assisted ventilation at day 28 or dies prior to day 28, VFD will be zero (0).

Unassisted breathing is defined as breathing with a face mask or nasal prong oxygen (or room air) following extubation, T-tube breathing, breathing with continuous positive pressure (CPAP $\leq$ 5 cm H_2O), or tracheotomy mask breathing.

VASOPRESSOR USAGE

Vasopressor usage will be determined by:

1. Length of vasopressor treatment until day 28 or ICU discharge whichever comes first.
2. Time on more than a single vasopressor until day 28 or ICU discharge whichever comes first.

INFECTION:

Infection will be defined as "a positive body fluid (blood, sputum, urine, etc.), tissue (muscle, skin, etc.), or indwelling device (catheter tip, pacer lead, etc.) culture from non-contaminant pathogen." Infection rate is the total number of infection episodes up to hospital discharge or day 60 (whichever comes sooner) per group.

HOSPITAL LENGTH OF STAY:

Both ICU and Hospital length of stay will be defined based on the ICU and acute hospital admissions during which the patient was randomized and enrolled in the SCD003 Trial. Length of stay will be evaluated on the basis of the mean number of days of ICU/hospital stay following randomization into the SCD003 trial. Hospital discharge will be defined as discharge from acute care, whether to acute rehabilitation, transitional care, long term care or home.

DURATION OF RENAL SUPPORT:

The duration of renal support will be defined as the numbers of days from the initiation of renal replacement therapy to final dialysis treatment. Duration of renal support will be censored if the patient is still dialysis dependant at the time of death.

RECOVERY OF RENAL FUNCTION:

Recovery of renal function will be defined as lack of need for continuous dialysis support, and will be classified as complete recovery, partial recovery, or no recovery.

Complete recovery of renal function will be defined as serum creatinine that is no more than 0.5 mg/dL greater than baseline; partial if the level remained above 0.5mg/dL above the baseline but not dialysis dependant. Patients who remain dialysis dependant at study completion or time of death will be categorized as having no recovery of renal function.

SCD-ARF INTEGRITY:

Selective Cytopheretic Device integrity is defined as use of the SCD-ARF without failure. The SCD-ARF will be evaluated for the presence of clotting, leaking or any other malfunction.

APPENDIX E
CRRT MANAGEMENT

Continuous Renal Replacement will be provided to all patients randomized in the SCD-003 Trial using automated equipment with integrated ultrafiltration control. All CRRT equipment will be provided to each participating center by the Sponsor.

CRRT MANAGEMENT

CRRT Modality:	Continuous
Blood Flow Rate:	Prescribed by Investigator Team – Treating Team
Dialysate Type:	Prescribed by Investigator Team – Treating Team
Dialysate Rate:	Prescribed by Investigator Team – Treating Team
Replacement Fluid Type:	Prescribed by Investigator Team – Treating Team
Ultrafiltration:	Prescribed by Investigator Team – Treating Team
Anticoagulation:	Regional Citrate Anticoagulation Only
Anticoagulation Prescription:	Prescribed by Investigator Team – Treating Team
CRRT Device	B.Braun Diapact – Provided by Sponsor
Hemofilter:	Fresenius Polysulfone® F50NR – Provided by Sponsor
CRRT System Change:	Per participating center protocol
SCD Change:	Every 24 hours or as needed due to clotting
CRRT or CRRT+SCD Interruption:	Up to six (6) hours per 24 hour period

APPENDIX F
CHILD-PUGH LIVER STAGING

Parameters:

(1) Serum total bilirubin
(2) Serum albumin
(3) Prothrombin time
(4) Grade of hepatic encephalopathy
(5) Ascites

PARAMETER	FINDING	POINTS
**Total Bilirubin**	< 2 mg/dL	1
	2 – 3 mg/dL	2
	> 3 mg/dL	3
**Serum Albumin**	> 3.5 g/dL	1
	2.8 – 3.5 g/dL	2
	< 2.8 g/dL	3
**Prothrombin Time (seconds) Prolonged**	1 – 4 sec prolonged	1
	4 – 6 sec prolonged	2
	> 6 sec prolonged	3
**Grade of Hepatic Encephalopathy**	None	1
	Grade 1 or 2	2
	Grade 3 or 4	3
**Ascites**	None	1
	Mild	2
	Severe Tense	3

Total score = (bilirubin score) + (albumin score) + (PT score) + (encephalopathy score) + (ascites score)

Interpretation:

GRADE	TOTAL SCORE	RISK
A	5 or 6	Good
B	7–9	Moderate
C	10–15	Poor

Minimum score: 5; maximum score: 15

APPENDIX G
SCD-003 Study Schedule of Events

	Baseline	Hr 0-24	Hr 25-48	Hr 49-72	Hr 73-96	Hr 97-120	Hr 121-144	Hr 145-168	Therapy End
**Vital Signs/Physical Assessment**									
Temperature , Body Weight	X	X	X	X	X	X	X	X	
Blood Pressure, Heart Rate	X	q4h	q4h	q4h	q4h	q4h	q4h	q4h	
EKG	X								
Sequential Organ Failure Assessment (SOFA Score)	X	X	X	X	X	X	X	X	
Physical Exam	X								
Urine Output	X	X	X	X	X	X	X	X	
Net fluid balance (24 hours)		X	X	X	X	X	X	X	
**Clinical Laboratory Testing**									
CBC with differential	X	q8h	q8h	q8h	q8h	q8h	q8h	q8h	
BUN/Creatinine	X	X	X	X	X	X	X	X	
Na, K, Cl, HCO_3, Ca, Mg, PO_4, Ionized Calcium	X	X	X	X	X	X	X	X	
Haptoglobin	X								X
ALT, AST, LDH, Bilirubin, ALP, TP, Albumin, Glucose	X	X	X	X	X	X	X	X	
PT, PTT, INR	X	X	X	X	X	X	X	X	
Urinalysis (if urine is available)	X								
**Respiratory**									
Arterial Blood Gas (pH, PaO_2, $PaCO_2$) (if on vent)*	X	q4/q12h	q12h	q12h	q12h	q12h	q12h	q12h	
Ventilator Settings (Mode, FiO_2, TV, PEEP, RR, NO)	X	q4/q12h	q12h	q12h	q12h	q12h	q12h	q12h	
Non-ventilator Settings (Face mask, supplement O_2, etc)	X	q4/q12h	q12h	q12h	q12h	q12h	q12h	q12h	
**Blood For Research**									
Blood for Biomarkers	X	X (hr24)		X (hr 72)		X (hr 120)		X (hr 168)	X
**Extracorporeal Device Parameters**									
CRRT (Settings)	X (hour 0)	q6h	q6h	q6h	q6h	q6h	q6h	q6h	
Post Filter Ionized Calcium	X (hour 0)	q6h	q6h	q6h	q6h	q6h	q6h	q6h	
CRRT Device and SCD-ARF Performance (Event Driven)		X	X	X	X	X	X	X	
**Other (Event-Driven)**									
Adverse Events	X	X	X	X	X	X	X	X	X
Concomitant Medications, Blood products	X	X	X	X	X	X	X	X	X
Diagnosis/Therapeutic Procedures	X	X	X	X	X	X	X	X	X
Microbiology/culture data	X	X	X	X	X	X	X	X	

*Time points are different depending on the presence of an A-line; see section 6.3 and 6.4 for details

APPENDIX H
SCD-003 Study Schedule of Events: Follow-up Period
Day 1 - 7 or until ICU discharge (whichever occurs first unless otherwise specified)

Measurement	Hour 0 - 24	Hours 25 – 168[1]
**Vital Signs/Physical Assessment**		
Blood pressure and heart rate	X	X
SOFA Score (daily until ICU discharge or Day 28)	X	X
Physical Exam	X	
Body weight and temperature	X	X
EKG	X	
Urine Output (from previous 24 hours)	X	X
Net Fluid Balance – (from previous 24 hours) (until ICU discharge or Day 28)	X	X
**Clinical Laboratory Tests**		
CBC with differential	X	
BUN/Creatinine	X	
Na, K, Cl, HCO_3, Ca, Mg, PO_4, Ionized Calcium	X	
**Respiratory:**		
Arterial Blood Gas (pH, PaO_2, $PaCO_2$)[2]	X	X
Ventilator Settings (Mode, FiO_2, TV, PEEP)	X	X
Non-ventilator Settings (Face mask, supplement O_2)	X	X
**Other** (event driven)		
Renal Replacement Therapy status	X	X
Medications given	X	X
Pressor Usage (until ICU discharge or Day 28)	X	
Procedures performed	X	X
Adverse events	X	X
Microbiology/Culture Data (until Hospital Discharge or Day 60)	X	X

[1]Events listed in this column are to be performed once per 24 hour time period
[2]If on vent with an arterial line

APPENDIX I
SCD-003 Study Schedule of Events: Day 28 and Day 60

Measurement	Day 28[1]	Day 60[2]
Blood pressure, heart rate and temperature (if known)	X	X
Presence of mechanical ventilation (since last visit)	X	X
Presence of renal replacement therapy (since last visit)	X	X
SOFA (if still in ICU)[3]	X	
Fluid Balance (if still in ICU)[3]	X	
Pressor Usage (if still in ICU)[3]	X	
Microbiology/Culture Data (if still in Hospital)[3]	X	X
Hospital and ICU Discharge information	X	X
Adverse events	X	X
Serum Creatinine (if office visit takes place)	X	X
Quality of Life Questionnaire		X

[1] This visit may be done via telephone call or office visit. Please record the above information on day 28 (+7 days) following enrollment.

[2] This visit may be done via telephone call or office visit. Please record the above information on day 60 (+7 days) following enrollment.

[3] This refers to the original Hospital and ICU admissions

APPENDIX J

INFORMED CONSENT TEMPLATE

A Multi-Center, Randomized, Controlled, Pivotal Study to Assess the Safety and Efficacy of A Selective Cytopheretic Device (SCD) In Patients with Acute Kidney Injury (AKI)

Informed Consent Statement

STATEMENT OF RESEARCH

You, or your family member, may be eligible to take part in a research study using a new medical device called the Selective Cytopheretic Device (SCD). You may decide to be in the study or decide not to take part at all. It is your decision. If you decide to be in the study, you may withdraw or stop the study at any time. No matter what you decide, your decision will not lead to any penalty or affect your regular medical care from the doctors and hospital staff. This document describes what will be done to you should you decide to participate and any risks that might be involved in this study. It is important that you read the following information and ask as many questions as necessary to be sure you understand what you will be asked to do.

INFORMATION ABOUT AKI AND THE STUDY DEVICE

You, or your family member, have been diagnosed with a very serious and often deadly disease known as acute kidney injury (AKI). AKI is a condition where the kidneys are not able to make enough urine. Therefore another way to remove waste from your body is needed. One way of removing waste from the body is called Continuous Renal Replacement Therapy (CRRT). There are different types of CRRT, but all allow waste to be removed from your body because your kidneys can not do this any longer. Your doctor has recommended this therapy for you or your family member. You may already be receiving CRRT or you are about to begin.

Acute kidney injury can also cause your immune system to not work correctly. Your immune system usually helps your body get better, but having an immune system that doesn’t work may be one of the reasons that AKI is such a morbid and deadly condition. White blood cells, which are key parts of the immune system, are overactive in AKI and may be the reason your immune system isn’t working the way it should.

While we don’t know the exact reason the device in this study, the SCD, may help you, it is thought it binds and stops overactive white blood cells. When the white blood cells are in the SCD they come in contact with a medicine called citrate. Citrate is sometimes used in patients that need CRRT. It is an anticoagulant, meaning that it stops your blood from clotting while it is going through the CRRT machine. Should you choose to be a part of this study, your doctors will use citrate as part of your CRRT treatment whether you are treated with the device or not.

If you decide to participate, you will be placed into one of two study groups. Half (50%) of the subjects in the entire study will placed on the SCD and the other half will not. Whether you will be treated with the SCD is completely random (like flipping a coin).

Your doctors and other caregivers do not decide which group you will be in. However, you will know which group you are in when the study tests begin.

PURPOSE OF RESEARCH AND LENGTH OF PARTICIPATION IN STUDY

The purpose of this study is to test if the SCD helps someone with AKI recover faster than without it.

You will remain in the study for 60 days after you begin the study unless you choose to stop before that.

Up to 344 subjects will take part in this study at up to 30 centers.

THIS IS WHAT WILL HAPPEN IF YOU DECIDE TO BE IN THIS RESEARCH STUDY

Should you decide to participate, your doctor or a member of the study team will ask you to sign this informed consent document. They will then talk to you about your disease and ask you questions about your health. They will determine if you meet the conditions for the study. It is possible that after looking at your medical history or your current condition, they may discover that you are not able to join the study. Some studies have specific requirements for being a part of the study. If this happens, no data will be collected from you and your healthcare treatment will continue as if you were never asked.

Beginning of study:
If the study team believes you are able to be a part of this study, they will enroll you into the study and you will be put into the SCD (study) group or the non-SCD (control) group by a completely random process that the medical staff has no control over. No matter which group you are in, medical information about you will be collected for the study. The study team will look at your medical records, complete a physical exam and EKG, and a few blood tests will be performed. An EKG is a test that shows the doctors how your heart is beating. Most of the blood tests that will be done are routine blood tests that are done on someone with AKI, but we will also be taking a small amount of blood that will be sent to an outside laboratory and tested for different biomarkers of kidney injury. A biomarker is something that shows up in the blood that may indicate a health problem. This could help future patients with acute kidney injury.

Observation/Treatment Period:
If you are in the SCD group, the study device will be connected to the CRRT tubing so there are no extra needle sticks or extra tubes that need to be inserted in your body. At all times while the study device is running trained medical personnel will be at your bedside to make sure the SCD is working properly. The study device may be connected for as long as 7 days. It will be stopped earlier if your kidneys have recovered, your doctor thinks it is necessary, you decide that you longer wish to participate or if there is any evidence that the device is not working properly.

If you are in the control (non-SCD) group, you will be placed on the same CRRT machine, however there will be no SCD device connected to you.

In all patients, certain tests will be performed during the time you are on the study device or on CRRT (up to 7 days). They include things like taking your blood pressure and temperature, and collecting information about your general medical condition and what medications you are receiving. Blood tests will also be performed daily. Some blood tests are tests that are normally done on someone with AKI or are done for safety reasons. We will also be taking a small amount of blood that will be sent to an outside laboratory and tested for different biomarkers. This will allow us to see how the SCD is working.

Follow-up Period:
After you are taken off CRRT or you have been on CRRT for 7 days, you will remain in the study until you have been in the study for 60 days, even if you are discharged from the hospital.

In the first 7 days after the observation/treatment period, information will be collected regarding your medical condition and what medications you have been given. An EKG, physical exam and blood tests will also be done on the first day after the observation period. These tests are typical of a patient in the hospital with AKI. If you remain in the ICU after this time, additional blood tests and clinical information may be collected.

The final follow-up study visits will occur at 28 days following the start of the study and again at 60 days following the start of the study. The study team may have you come back to the hospital or office for this visit, or they may contact you by telephone. They will ask you questions about your health since the last visit. If you are in the doctor's office or hospital, they will collect some vital signs and possibly do a blood test to see if your kidneys are working properly.

You will also be asked to complete a short questionnaire towards the end of the study. There will be questions asking you about how you feel and how your health is affecting your everyday activities. You do not have to answer any questions that you don't want to. The study staff may have you mail the form back in or may ask you to complete it in person or over the phone.

The research staff will review your medical records from the time you were in the study. After your 60 day visit, you will no longer be part of the study and no more information will be collected from you or your medical records.

BENEFITS

There is no guarantee that you may receive any benefit from participation in this study. If you are in the SCD group, the SCD may help resolve or moderate your disease.

While there may not be a direct benefit to you for participation in this study, the knowledge we will learn may benefit others in the future. The information gathered in this study might also benefit people with AKI in the future.

RISKS

Any experimental or test therapy is associated with risk. Some of these risks are not known.
Possible side effects with the SCD is a decrease in your blood pressure, a decrease in the number of white blood cells and platelets in your blood (platelets are cells that help your blood clot), or bleeding. In some cases any of the side effects could be life threatening. You will be closely monitored by the study team and your regular hospital caregivers.

This product has not been tested in pregnant females. Therefore, risks to an embryo or fetus are unknown at this time. You should not be pregnant or intend to become pregnant while taking part in this study. If you are a woman of child bearing potential, a pregnancy test will be performed before you enter the study. If you suspect that you have become pregnant during the study, you must notify the study doctor immediately.

There is a risk of blood clotting in the SCD or CRRT tubing. To prevent blood clotting, regional citrate anticoagulation is used in the study. Citrate is not toxic and is also produced in small amount in the human body.

A rare, potentially serious risk would be severely low or high blood calcium levels. This could happen if you receive too much calcium, citrate, or dialysis solution. This risk is lowered by the close monitoring of the citrate procedure. In addition, your blood will be tested for calcium levels.

There are risks associated with the blood draw. It may be associated with discomfort, bruising, infection at the site and formation of a blood clot. All efforts will be made to collect these samples at the same time as your routine (non-study) blood draws. Also, if you have an arterial line in place, blood may be collected from there.

There is a risk of the loss of confidentiality. The researchers will make every effort to keep your medical records safe and you will have a study number that will be used to identify you.

Participating in this study could cause you some inconvenience.

There may be additional risks or discomforts that are not known at this time.

STOPPING EARLY

If you are in the SCD group, you may decide to stop being treated with the SCD at any time. If you decide to stop the SCD treatment, we may ask you to continue in the follow-up period to gather safety information on you. However, you may also decide to leave the study completely.

If you leave the study before the 60 days, there will be no penalty to you. You will not lose any benefits to which you may otherwise be entitled.

All information collected up to that point will remain in the study, but no further information will be collected. If you choose to tell the researchers why you are leaving the study, your reasons for leaving may be kept as part of the study record.

Your doctor may also decide to remove you from the study if you don't follow instructions or your condition changes while you are taking part in the study and the doctors feel that it is not in your best interest to stay in the study. You may also be taken out of the study early if the study is suspended or canceled.

If you leave the study early for any reason, your doctor may ask you to return for one or more study visits to complete the final study activities for safety reasons.

Any significant new research findings regarding the study device that may affect your willingness to participate or continue in this study will be provided to you as updated information.

WHAT OTHER OPTIONS ARE THERE?

You do not have to participate in this study and can continue with your usual AKI treatments. Talk to your doctor about your choices before you decide if you will take part in this study.

WILL IT COST TO PARTICIPATE?

We do not expect there to be any additional costs to you if you participate in this study. You or your health plan will pay for all the things you would have paid for even if you were not in the study, including the ICU stay, CRRT, and other hospital related health costs. The SCD study device will be provided by the sponsor at no cost to you or your health insurance provider.

WILL I BE PAID TO PARTICIPATE?

There is no payment made or financial benefit to you for taking part in this study. You will also not receive any money or other benefits as a result of any products, procedures, or other items developed from the results of this study.

WHAT IF I AM INJURED?

If you are injured as a direct result of being in this study and not due to the natural course of an underlying disease or treatment process, necessary medical care will be provided to you. It is possible that the costs of this medical care may be billed to your insurance company. However, if your insurance company does not pay these costs or if you do not have insurance, the study sponsor, CytoPherx, Inc. will cover the costs of your medical treatment necessary to diagnose and/or treat your injury. CytoPherx, Inc. has no plans to pay for such things as lost wages, disability, or discomfort due to the injury. No other compensation is routinely available. You are not giving up any of your legal rights by signing this consent form.

CONFIDENTIALITY

Although every reasonable effort will be made to protect the confidentiality of your records, such protection cannot be guaranteed. We shall put the information collected about you during the study into a research record. This research record will not show your name, but will have codes entered in it that will allow the information to be linked to you. Your medical information will be kept confidential by the study doctor and staff and will not be made publicly available unless disclosure is required by law.

We may collect and use:

- Your existing medical records
- New health information created during this study
- Health insurance and billing information (a separate billing release authorization may be required, but is not necessary for participation)

We may release this information to the following people:

- The Principal Investigator and his/her associates who work on, or oversee the research activities
- The research sponsor company, CytoPherx, Inc, it's Contract Research Organization (CRO), and any authorized consultants
- Government officials who oversee research

Data and information obtained from this study that does not identify you individually may be published in medical journals or presented at medical conferences. By signing this consent form, you authorize the record review, information storage, and data transfer described above.

Once your information has been released according to this consent form, it could be released again and may no longer be protected by federal privacy regulations.

This consent form, test results, medical reports and other information about you from this study may be placed into your medical record. Generally, you are allowed to look at your medical record.

This consent to use and release your personal and health information will expire at the end of this research study.

You do not have to sign this consent to release your medical information and may cancel it at any time. If you decide not to sign this consent or cancel your consent, you cannot participate in this study. If you notify us that you wish to stop participating in this study, we may continue to use and release the information that has already been collected. To cancel your consent, send a written and dated notice to the principal investigator at the address listed on the first page of this form.

A description of this clinical trial will be available on http://www.ClinicalTrials.gov, as required by U.S. Law. This Web site will not include information that can identify you. At most, the Web site will include a summary of the results. You can search this Web site at any time.

FUTURE BLOOD TESTING

Researchers in this study are dedicated to researching biomarkers of kidney disease related to AKI. The data and blood samples collected during this study are important to this study and to future AKI research. With your additional consent, blood collected during this study (at the start of treatment and during the observation period) will be kept in an outside laboratory for an indefinite period of time with samples from other subjects who have consented to be in this study. All personally identifying information will be removed from the samples. They will be identified only by a unique study number.

These samples may be used in the future in ongoing research related to biomarkers involved in AKI and other kidney conditions. They will only be studying kidney disease, which you have already been diagnosed as having. They will not be researching non-kidney related disease.

These samples will be sent an outside laboratory chosen by the sponsor, CytoPherx, Inc, but we will not give them any information that would personally identify you. We will not put the results of any tests conducted on these samples in your medical record. **You may still participate in this study even if you do not agree to participate in the sample repository.**

[ ] _____________ (check box and initial) I AGREE to allow my blood samples to be saved for future biomarker studies.

[ ] _____________ (check box and initial) I DO NOT AGREE to allow my blood samples to be saved for future biomarker studies. (Please note that your blood will still be sent out and tested for the current biomarkers as explained above, but your samples will not be saved for future testing).

WHO DO I CALL WITH QUESTIONS ABOUT THE STUDY OR TO REPORT AN INJURY?

________________ MD or his /her staff member has explained this research study and has offered to answer any questions. If you have questions about the study procedures, or to report an injury you may contact _______________MD at telephone number ________________.

CONSENT

Volunteer's Statement

- I voluntarily agree to participate in this study.
- I understand that the study sponsor, CytoPherx Inc. may stop the study at any time. If this happens, I will no longer receive the study treatment and planned evaluations.
- I have read and understand this statement of informed consent and the risks described.
- I understand that I will receive a signed and dated copy of this consent form.
- I understand that I may withdraw my consent at any time.
- I have had a chance to ask questions and understand the answers given to all of my questions.

____ __________________
Signature of Study Volunteer *Date*

____
Printed Name of Volunteer

OR

____ __________________
Signature of Legally Authorized Representative *Date*

____
Printed Name of Legally Authorized Representative

____ __________________
Signature of Person obtaining Informed Consent *Date*

____
Printed Name of Person obtaining Informed Consent
